# Whole Genome Sequencing Identifies a Deletion in Protein Phosphatase 2A That Affects Its Stability and Localization in *Chlamydomonas reinhardtii*

**DOI:** 10.1371/journal.pgen.1003841

**Published:** 2013-09-26

**Authors:** Huawen Lin, Michelle L. Miller, David M. Granas, Susan K. Dutcher

**Affiliations:** 1 Department of Genetics, Washington University School of Medicine, St. Louis, Missouri, United States of America; 2 Center for Genomic Sciences and System Biology, Washington University School of Medicine, St. Louis, Missouri, United States of America; Rutgers University, United States of America

## Abstract

Whole genome sequencing is a powerful tool in the discovery of single nucleotide polymorphisms (SNPs) and small insertions/deletions (indels) among mutant strains, which simplifies forward genetics approaches. However, identification of the causative mutation among a large number of non-causative SNPs in a mutant strain remains a big challenge. In the unicellular biflagellate green alga *Chlamydomonas reinhardtii*, we generated a SNP/indel library that contains over 2 million polymorphisms from four wild-type strains, one highly polymorphic strain that is frequently used in meiotic mapping, ten mutant strains that have flagellar assembly or motility defects, and one mutant strain, *imp3*, which has a mating defect. A comparison of polymorphisms in the *imp3* strain and the other 15 strains allowed us to identify a deletion of the last three amino acids, Y_313_F_314_L_315_, in a protein phosphatase 2A catalytic subunit (PP2A3) in the *imp3* strain. Introduction of a wild-type HA-tagged PP2A3 rescues the mutant phenotype, but mutant HA-PP2A3 at Y_313_ or L_315_ fail to rescue. Our immunoprecipitation results indicate that the Y_313_, L_315_, or YFLΔ mutations do not affect the binding of PP2A3 to the scaffold subunit, PP2A-2r. In contrast, the Y_313_, L_315_, or YFLΔ mutations affect both the stability and the localization of PP2A3. The PP2A3 protein is less abundant in these mutants and fails to accumulate in the basal body area as observed in transformants with either wild-type HA-PP2A3 or a HA-PP2A3 with a V_310_T change. The accumulation of HA-PP2A3 in the basal body region disappears in mated dikaryons, which suggests that the localization of PP2A3 may be essential to the mating process. Overall, our results demonstrate that the terminal YFL tail of PP2A3 is important in the regulation on *Chlamydomonas* mating.

## Author Summary

Whole genome sequencing is a powerful tool to detect changes in genomic DNA. However, how to identify a causative mutation from over 20,000 changes remains a big challenge. For the unicellular green alga *Chlamydomonas*, we built a library that consists of over 2 million changes from 16 strains. A comparison of changes found in a mutant strain with a mating defect, *imp3*, to 15 other strains, leads to the identification of a three amino acid deletion in the catalytic subunit of a protein phosphatase 2A (PP2A3). The mating defect of *imp3* is rescued by an HA-tagged *PP2A3* gene. Introduction of the HA-tagged *PP2A3* gene with various mutations in these three amino acids reveals that they play a key role in stabilizing and ensuring the proper localization of PP2A3. The ubiquitous enzyme PP2A is involved in diverse cellular processes. Our discovery that PP2A3 is involved in the *Chlamydomonas* mating signaling pathway, which also contains the polycystin2 homolog (PKD2), makes *Chlamydomonas* mating an excellent model to study ciliary/flagellar signaling. Since both PP2A and PKD2 play important roles in human health, further investigation of the relationship between these two proteins in *Chlamydomonas* will facilitate better understanding of their functions.

## Introduction

Forward genetics allows the identification of mutants with phenotypes of interest and the mechanistic understanding of biological processes [1], [2]. While gene lesions generated by insertional mutagenesis can be identified by Southern blot analysis [3]–[6] or PCR-based approaches [7]–[9], identification of mutations induced by radiation or chemical mutagenesis rely on time-consuming meiotic mapping [10]–[14]. Recently, single nucleotide polymorphism (SNP) discovery by whole genome sequencing (WGS) provides a faster and more efficient method to identify causative mutations [15]–[18]. However, in model organisms such as *Arabidopsis thaliana*, the number of SNPs can vary from 2,000 to 900,000, depending on the strain background [19]. In *Caenorhabditis elegans*, the number of SNPs between two strains is ∼100,000 [16]. In the unicellular biflagellate green alga *Chlamydomonas reinhardtii*, ∼38,000 SNPs were identified in individual mutant strains [18]. Therefore, identification of the causative SNP from a large number of SNPs remains a challenge.

In *Chlamydomonas*, a model organism for the study of flagellar function, photosynthesis, biofuels, and sex determination, the causative genes in several hundred mutant strains generated by radiation or chemical mutagenesis remain unidentified [20]. UV mutagenesis mutant screens generated 12 impotent (*imp*) mutant strains that have either abolished or reduced mating efficiency [21], [22]. In *Chlamydomonas*, the sex of a cell is controlled by two alleles, *plus* or *minus*, at the mating-type (*MT*) locus [23]. The differentiation from exponentially growing vegetative cells to gametes is triggered by nitrogen starvation via an unknown mechanism. When gametes of the opposite mating-types are mixed together, they agglutinate via flagellar membrane-associated proteins, agglutinins, to trigger a mating signal transduction pathway. This signal cascade leads to cell fusion and the formation of zygotes. Among the previously characterized *imp* mutant strains, *imp2*, *imp5*, *imp6*, *imp7*, and *imp9* are allelic and encode SAG1, which is the *plus* agglutinin [24]. The *imp10* and *imp12* mutant strains encode SAD1, the *minus* agglutinin [24]. The *imp8* strain is defective in O-glycosylation and is allelic with the *GAG1* locus [25]. The *imp1* and *imp11* mutant strains map within the *MT* locus [26]–[29] and carry mutations in *FUS1* in *plus* and *MID1* in *minus* cells, respectively. Only *imp3* and *imp4* remain unidentified among the original collection of impotent mutants. Unlike the other *imp* strains that abolish mating (<1%), the mating efficiency of *imp3* and *imp4* strains varies from 10% to 50%, in contrast to >80% in wild-type cells 1 hour after mixing of the gametes. Neither mutation is linked to the *MT* locus or to the other [21]. Saito *et al.* [30] suggested that activation of uncharacterized flagellar adenylate cyclases is blocked in *imp3* cells, and IMP3 is required in the mating signaling pathway. However, the causative genes in *imp3* and *imp4* remain unidentified due to their partial, weak mating phenotype and the difficulty to map this phenotype.

Serine/threonine phosphorylation is generated by 300–400 kinases but is reversed by a relative small number of phosphatases. The serine/threonine phosphatase, protein phosphatase 2A (PP2A), plays an important role in signaling pathways. It is a ubiquitous enzyme that is involved in diverse cellular processes; they include cell cycle control, cell growth, microtubule stability, and signaling [31]. The PP2A heterotrimeric holoenzyme contains 3 subunits; they are a catalytic subunit (C subunit), a scaffold subunit (A subunit), and a regulatory subunit (B subunit). The catalytic subunit (PP2Ac) is highly conserved across species and it shares significant sequence similarity to the PP4 and PP6 protein phosphatases [32]. The catalytic activity of PP2Ac can be modulated by post-translational modifications that include phosphorylation/dephosphorylation in the conserved C-terminal motif T_304_PDY_307_FL_309_ on T_304_ and Y_307_ and methylation/demethylation of L_309_ [33]. The scaffold subunit of PP2A contains multiple HEAT repeats that confer conformational flexibility to both the catalytic subunit and the regulatory subunit [32]. The regulatory subunit of PP2A falls into four distinct families, which are known as B (PR55), B′ (B56 or PR61), B″ (PR72), and B′″ (PR93/PR110). It is believed that different families of the B subunit target PP2A to different cellular locations and bind to different substrates [32], [33]. In *Chlamydomonas*, sequence similarity reveals four catalytic subunits, two scaffold subunits, and five regulatory subunits [34].

In this study, we took advantage of whole genome sequencing of 16 different wild-type and mutant strains to generate a SNP/indel library. SNP/indel comparison, in conjunction with meiotic mapping, allowed the quick identification of the causative mutation in the *imp3* mutant strain, which contains a C-terminal three amino acid deletion in the conserved TPDYFL motif of a PP2A catalytic subunit (PP2A3). The deletion of YFL affects not only the stability of PP2A3, but also the accumulation of PP2A3 around the basal body area.

## Results

### Construction of a *Chlamydomonas* SNP/indel library

A previous study using 101-bp paired-end Illumina sequencing of an *IFT80* mutant strain, NG30*/ift80*, revealed that over 38,000 SNPs/indels are present compared to the *Chlamydomonas* reference genome [18]. It was a challenge to identify the causative mutation from such a large number of changes. We reasoned that if changes are found in other unlinked mutant strains or in wild-type strains, they are not causal and could be eliminated from further analysis. Therefore, a collection of changes from multiple strains would be necessary to remove as many non-causative changes as possible to reveal the causative mutation in a given mutant strain.

To build a library of changes, we first sequenced four wild-type strains (CC-124, CC-125, isoloP (CC-4402), and isoloM (CC-4403)). In *Chlamydomonas*, the major laboratory strains CC-124 and CC-125 were first isolated from a single diploid zygote, 137c, in 1945 [35] and these strains have been used as the parents in many mutant isolations. CC-125 is also the background strain of CC-503, the strain used for the *Chlamydomonas* reference genome assembly. CC-124 carries a *minus* mating-type locus (*mt−*) and CC-125 is mating-type *plus* (*mt+*). The other two wild-type strains, isoloP (*mt+*) and isoloM (*mt−*), were generated by crossing CC-124 by CC-125 to obtain meiotic progeny. Several progeny from this cross that gave the fastest and highest percentage of mating were backcrossed to CC-124. This procedure was repeated ten times in an attempt to obtain isogenic strains that were named isoloP and isoloM. Sequencing of these wild-type strains identifies 13,000 to over 100,000 changes relative to the reference genome. We also sequenced is a highly polymorphic strain S1C5 (CC-1952) that is frequently used in meiotic mapping with molecular markers. Over 2 million changes are identified from this strain. In addition, since we were interested in identifying the causative mutations from a variety of mutant strains, we also performed whole genome sequencing on ten mutant strains that were generated by either chemical or UV-mutagenesis. Five of them (*fla18*, *fla24*, *fla9*, *uni1*, *ift80*) have flagellar assembly defects, four (*ida3, pf23, pf7, pf8*) have motility defects, and one has a mating defect (*imp3*). One additional mutant strain, *cnk10*, which has a flagellar assembly defect, was generated by insertional mutagenesis of the CC-125 strain (Lin and Dutcher, unpublished). The number of changes in individual mutant strains varies from 22,000 to over 150,000 (Table 1). The sequencing coverage of individual strains ranges from 26× to 162× (Table 1). Overall, 2,557,197 changes are included in this SNP/indel library and it is available at http://stormo.wustl.edu/SNPlibrary/.

**Table 1:** Strains used to generate a *Chlamydomonas* SNP/indel library.

Strain	# of SNPs/indels	% of Unique SNPs/indels	Total reads	Aligned reads	Unique aligned reads	Coverage	% Aligned	% Unique aligned	Read length^a^	Gene	Reference
**CC-124**	100737	n/a	51348146	49305462	48733566	41	96.0	94.9	PE101	n/a	
**CC-125**	13218	n/a	63165980	57295796	56394014	48	90.7	89.3	PE 101	n/a	
***cnk10***	85168	2	102235654	98762717	85305094	83	96.6	83.4	PE 101	*CNK10*	Lin *et al.*, manuscript in preparation
***isoloM*** b	65437	n/a	31985156	30876532	24823571	26	96.5	77.6	SE 36	n/a	
***isolo P*** b	56734	n/a	35971907	34756594	27826205	29	96.6	77.4	SE 36	n/a	
**S1C5**	2357347	n/a	63454170	42757481	36454703	36	67.4	57.5	PE 101	n/a	
***fla18*** c	1537589	2	157313244	111212684	103801048	94	70.7	66.0	PE 101	*FLA10*	Dutcher *et al.*, submitted
***fla24***	72298	1	63540680	60545846	59645402	51	95.3	93.9	PE 101	*DHC1b*	Dutcher *et al.*, submitted
***fla9***	83402	2	175939240	171264758	150672528	144	97.3	85.6	PE 101	*IFT81*	Dutcher *et al.*, submitted
***ida3***	84908	2	50333828	47285044	46531024	40	93.9	92.4	PE 101	Unknown	
***ift80***	39294	13	189425894	180695427	157888352	152	95.4	83.4	PE 101	*IFT80*	[18]
***imp3***	91066	7	200542622	192616309	166332474	162	96.0	82.9	PE 101	*PP2A*	This study
***pf23***	22198	11	173224614	166875062	148005238	140	96.3	85.4	PE 101	Unknown	
***pf7***	67691	2	113958070	106694994	104986866	90	93.6	92.1	PE 101	*CCDC40*	Dutcher *et al.*, manuscript in preparation
***pf8***	73576	1	104812850	97145742	95124272	82	92.7	90.8	PE 101	*CCDC39*	Dutcher *et al.*, manuscript in preparation
***uni1***	151608	21	49735180	48574834	41374522	41	97.7	83.2	PE 60	n/a	Siflow *et al.*, pers. comm.^d^
**Total**	2557197										
**Total exclude ** ***imp3***	2550105										

a PE, pair-end reads; SE, single-end reads.

b isoloM and isoloP were generated by 10 rounds of backcrosses of the wild-type strain CC124.

c The *fla18* strain sequenced was generated by a cross between *fla18* and S1C5 (CC-1952).

d pers. comm., personal communication.

**Figure 1:**
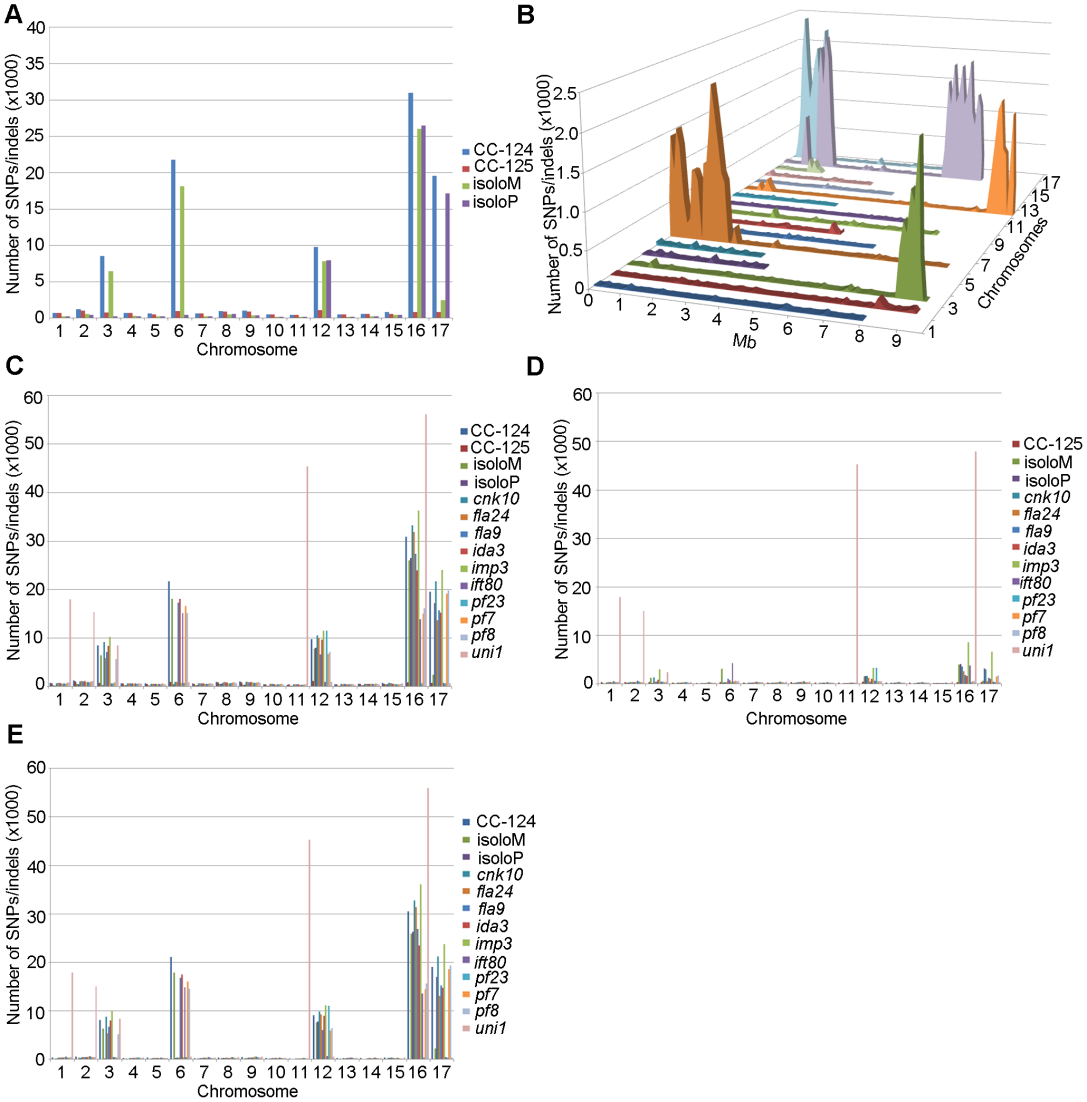
Distribution of SNPs/indels along the 17 *Chlamydomonas* chromosomes compared to the reference genome (v5.3). (A) Distribution of SNPs/indels in four wild-type strains (CC-124, CC-125, isoloP, and isoloM). (B) Distribution of SNPS/indels along the length of each chromosome in the CC-124 strain in Mb. The number of SNPs/indels is summed over each 100 kb interval. (C) Distribution in 14 strains used in this study, with the exception of *fla18* and S1C5. (D) Distribution in strains in part C after the subtraction of SNPs/indels found in the CC-124 strain. The *uni1* strain is the most divergent. (E) Distribution in 14 strains after the subtraction of SNPs/indels found in the CC-125 strain.

After collecting the SNPs/indels, we analyzed the distribution of the changes across the 17 chromosomes relative to the reference genome [36] (Figure 1). In the wild-type strain CC-125 (*mt+*), changes are spread evenly across all chromosomes from the reference genome (Figure 1A, Table 2). The wild-type strain CC-124 (*mt−*), which came from the same zygote as CC-125, contains 100,737 changes, which is about eight times the number of changes (13,218) found in CC-125. Around 90% of changes found in CC-124 are concentrated on five chromosomes: 3, 6, 12, 16, and 17 (Figure 1A, Table 2). A detailed analysis of numbers of SNPs/indels every 100 kb along the chromosomes in CC-124 reveals that the polymorphisms are not distributed evenly across these five chromosomes (Figure 1B). On chromosome 3, most SNPs/indels are between 8.5 Mb and 9.1 Mb. On chromosome 6, most SNPs/indels are within the first 1.9 Mb, which contains the *MT* locus. The mating-type locus is known to be polymorphic between the two sexes [37]. CC-124 carries the *MT minus* locus, which is not shared with the reference strain CC-503. On chromosome 12, most changes are observed between 9.0 Mb to 9.8 Mb. On chromosome 16, three distinct regions of SNPS/indels lie between 0.9–1.0 Mb, 1.5–2.0 Mb, and 6.4–7.8 Mb. A large number of changes within the 0.9–1.0 Mb were observed in a previous study of *Chlamydomonas* strains, *ift80* and *ac17* [18]. On chromosome 17, most changes are observed between 0.3 Mb to 1.5 Mb. The other two wild-type strains, isoloP (*mt+*) and isoloM (*mt−*), which are meiotic progeny of CC-124 and CC-125 after ten rounds of backcrosses to CC-124, were expected to show difference only on chromosome 6, which contains the *MT* locus. However, comparison of the sequence of these two strains indicates that they are not isogenic on chromosomes 3 and 17. The isoloM strain maintains large numbers of changes from the CC-124 parent on chromosome 3; isoloP contains large numbers of changes inherited from the CC-124 parent on chromosome 17 (Figure 1A, Table 2).

**Table 2:** Distribution of SNPs on all 17 chromosomes in wild-type strains.

Chromosomes	CC-124 (%)	CC-125 (%)	isoloM (%)	isoloP (%)
**1**	0.73	5.22	0.38	0.41
**2**	1.24	7.90	0.87	0.84
**3**	8.48	5.92	9.85	0.49
**4**	0.72	5.17	0.46	0.44
**5**	0.65	4.14	0.39	0.42
**6**	21.60	7.39	27.68	0.85
**7**	0.64	4.75	0.43	0.46
**8**	0.97	6.84	0.83	0.98
**9**	1.02	6.73	0.60	0.64
**10**	0.52	3.79	0.32	0.35
**11**	0.43	3.65	0.30	0.34
**12**	9.69	8.54	11.98	14.08
**13**	0.52	3.71	0.31	0.32
**14**	0.60	4.59	0.38	0.44
**15**	0.81	4.67	0.73	0.85
**16**	30.73	6.60	39.78	46.71
**17**	19.45	6.20	3.76	30.29

We performed the same analysis on the ten mutant strains, with the exclusion of *fla18*, because the sequenced strain came from a cross between the *fla18* mutant strain and S1C5. Ninety-six percent of *fla18* SNPs/indels are found in the S1C5 strain. Prior to Illumina sequencing, the ten strains were crossed to either CC-124 or CC-125 at least once. An accumulation of changes on chromosomes 3, 6, 12, 16, and 17 are observed in most strains (Figure 1C). To ask whether these changes are the same as found in CC-124, we subtracted SNPs/indels found in CC-124 from individual strains. The numbers of changes drop dramatically from 10,000∼35,000 to 1,000∼5,000 in almost all strains (Figure 1D). This suggests that changes in these strains are likely come from the CC-124 parent. In comparison, removal of CC-125 changes from these strains does not have an obvious effect on numbers of changes in these strains (Figure 1E). There is no correlation with the position of the causative mutant and an accumulation of SNPs along the chromosomes. One mutant strain, *uni1*, has a distinct distribution of changes along all chromosomes when compared to other strains (Figure 1C). Accumulation of changes is found on chromosomes 1, 2, 11, and 16. Removal of CC-124 or CC-125 changes from *uni1* has no significant effect on the distribution (Figure 1D and 1E). A comparison between 30,958 changes on chromosome 16 found in CC-124 and 56,192 changes on chromosome 16 found in *uni1* indicates that only 8,229 changes are common between these two strains. Thus, the SNPs/indels found in *uni1* have significantly different distribution than all other strains we analyzed. This mutant strain was generated from either strain 89 or 90 (CC-1009 or CC-1010) [38]. Pröschold *et al.* [39] categorized the common used laboratory *Chlamydomonas* strains into three basic sublines based on several criteria, including their ability to utilize nitrate, the mating-type locus, the number of rDNA repeats, and the presence of cell wall digestion metalloproteases (autolysins). Strains 89 and 90 belong to Subline II and CC-124 and CC-125 belong to Subline III. They have difference in all criteria described above. Thus it is not surprising to observe the difference of SNP/indel accumulations between *uni1* and 137c strains.

### Identification of the causative mutation in the *imp3* mutant strain

The 162× sequencing coverage of *imp3* reveal 91,066 changes in this mutant strain, which came from a 137c background. Comparison and subtraction between *imp3* and all other 15 strains finds 7,092 (7%) of these changes are unique to *imp3* (Table 1). Out of 954 changes in predicted exons, 145 are predicted to be synonymous changes and were excluded from further analysis. Changes vary from 1 to 297 on individual chromosomes (Table 3). In order to identify the causative mutation, we meiotically mapped the *imp3* mutant strain.

**Table 3:** Distribution of changes found in the *imp3* mutant strain.

Chromosomes	Total Changes	Changes Unique to *imp3*	Changes within coding regions	Synonymous changes excluded
**1**	725	151	2	1
**2**	1158	171	3	2
**3**	8409	879	181	158
**4**	715	97	2	2
**5**	621	80	1	1
**6**	18111	132	4	3
**7**	637	136	3	2
**8**	829	115	3	3
**9**	955	194	3	2
**10**	524	103	3	3
**11**	464	84	2	1
**12**	9725	495	54	40
**13**	524	107	3	2
**14**	580	96	3	2
**15**	614	53	2	2
**16**	23936	1979	354	297
**17**	15256	2160	329	286
**Total**	**91066**	**7092**	**954**	**809**

**Figure 2:**
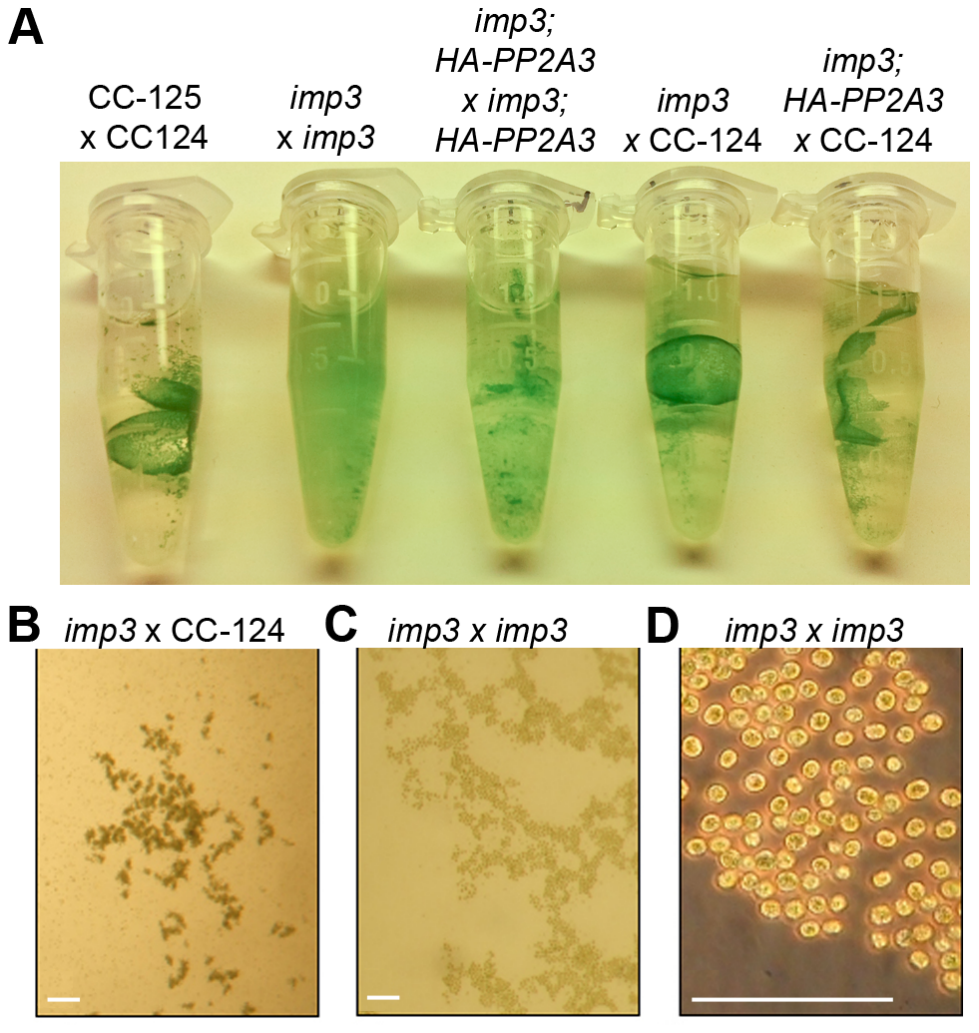
Pellicle phenotype after cell mating. (A) Pellicle formation after overnight mating between *plus* and *minus* gametes. Sheets of dark green pellicles are observed in matings between wild-type gametes (CC-125×CC-124), wild-type mated with either the untransformed (*imp3*×CC-124) or transformed *imp3* mutant strain (*imp3; HA-PP2A3*×CC-124), and between *imp3; HA-PP2A3* gametes. These pellicles are resistant to vortex or pipette disruption. In contrast, only small clumps of pellicles are found in the mating between *imp3* cells and they are easily disrupted by vortexing or pipetting. (B) Thick, dark green, multilayer pellicle found in mating between wild-type and *imp3* gametes. (C) Single layer pellicle that is light in color is found in matings homozygous for the *imp3* mutation. (D) Magnification view of cells in (C). Scale bar, 0.1 mm.

The *imp3* phenotype confers reduced mating efficiency, a phenotype that is challenging to analyze quickly. When wild-type gametes mate, the zygotes develop thick cell walls and form a dark green, multi-layered sheet of cells called the pellicle (Figure 2A). The mating between *imp3 mt+* and *imp3 mt−* do not form the thick pellicle sheet observed in wild-type cells or in mating between *imp3* and wild-type cells (Figure 2A). Observation under the dissecting microscope reveals dark thick multi-layer pellicle between *imp3* and wild-type gametes (Figure 2B). Mating between two *imp3* strains produces a single cell-layer pellicle that is light in color (Figure 2C and 2D). Individual progeny from 30 complete tetrads from a cross between *imp3* and wild-type cells were tested for this phenotype by mating with *imp3 mt+* and *imp3 mt−* tester strains. All 30 tetrads showed 2∶2 segregation of the single layer pellicle phenotype. This phenotype made it easy to distinguish between *imp3* and *IMP3* cells; this assay facilitated the molecular mapping of the *IMP3* locus.

**Figure 3:**
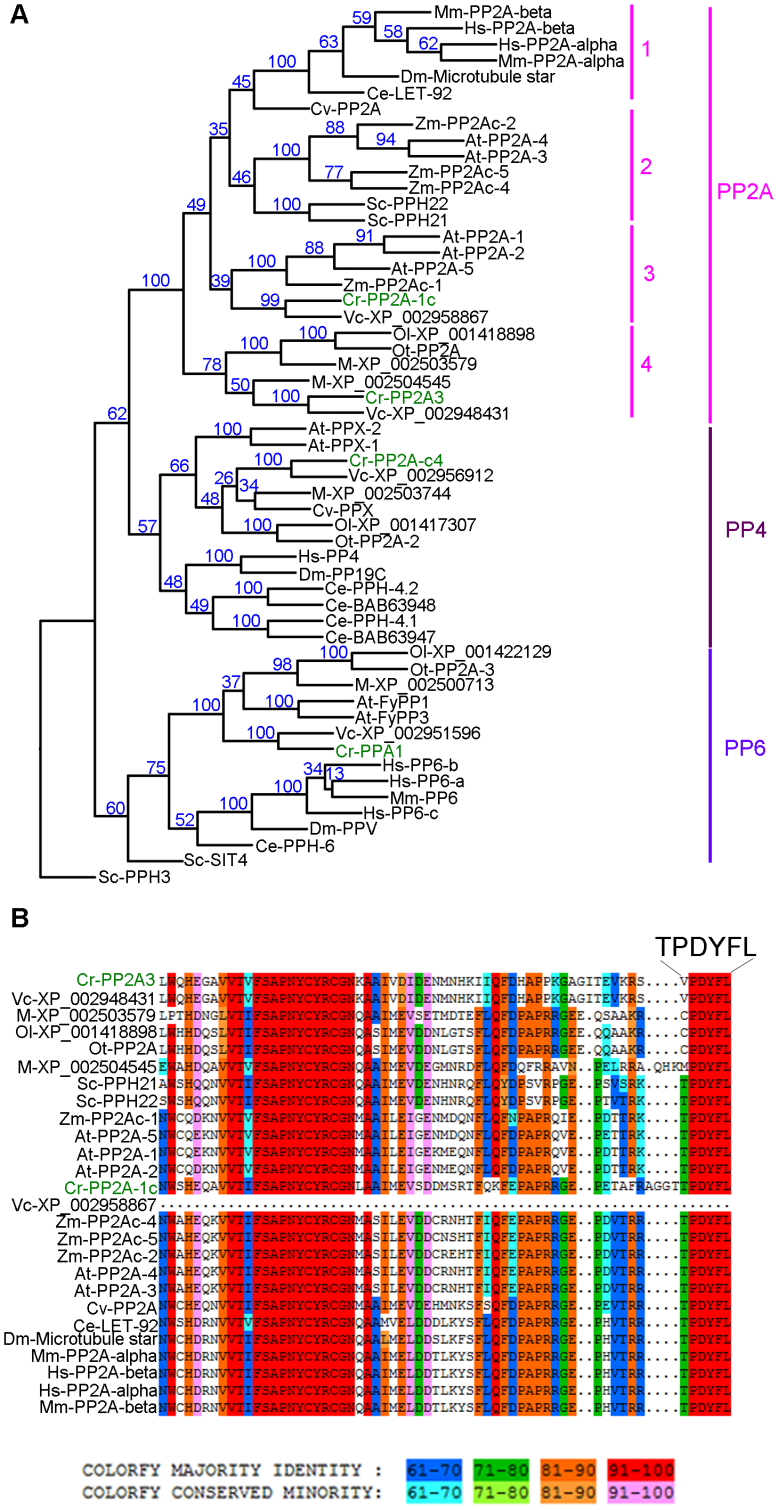
Phylogenetic analysis of PP2A, PP4, and PP6. (A) Phylogenetic analysis of 55 protein phosphatases from 13 organisms. Numbers next to each node were obtained from sampling of 100 bootstrap analyses. *Chlamydomonas* proteins are indicated in green. (B) Sequence alignment of the C-terminal 65 amino acids from all PP2A proteins defined by phylogenetic analysis in (A). The six terminal amino acids are shown in a larger font. The full alignment is shown in Figure S2. *Chlamydomonas* proteins are indicated in green. Sequence similarity percentage is displayed by colors shown below the alignment. Organism abbreviation: At, *Arabidopsis thaliana*; Ce, *Caenorhabditis elegans*; Cr, *Chlamydomonas reinhardtii*; Cv, *Chlorella variabilis*; Dm, *Drosophila melanogaster*; Hs, *Homo sapiens*; M, *Micromonas sp. RCC299*; Mm, *Mus musculus*; Ol, *Ostreococcus lucimarinus*; Ot, *Ostreococcus tauri*; Sc, *Saccharomyces cerevisiae*; Vc, *Volvox carteri*; Zm, *Zea mays*.

To map the *imp3* mutation, meiotic progeny from *imp3* crossed by the highly polymorphic strain S1C5 were obtained. Progeny from twenty tetrads that showed the single layer pellicle phenotype were mapped with dCAPS markers (Table S1). The single layer pellicle phenotype showed very tight linkage (18 parental: 0 recombinant) to the SCA8-2 marker, which maps to ∼6.6 Mb on chromosome 2 in a previous version of the genome assembly (v4) and maps to ∼3.5 Mb on chromosome 9 in the latest version (v5.3) of the genome assembly. To fine map the *imp3* mutation, additional progeny from over 100 tetrads were used for mapping. All three markers, 2-98, SCA8-2, and 55-193, which are about 17, 8, and 3 cM away from the *imp3* mutant, map to chromosome 2 in v4 genome assembly (Table S1). Both 2-98 and 55-193 are at ∼6.6 Mb and ∼7.1 Mb on chromosome 2 in v5.3 genome assembly, respectively (Table S1). Thus, we believe that genomic DNA with an unknown length around the SCA8-2 marker is misassembled in v5.3 genome assembly to chromosome 9 and the *imp3* mutation maps to chromosome 2. However, since we were unclear whether the *imp3* mutation is misassembled in v5.3 genome assembly, we examined polymorphisms in predicted exons on both chromosomes 2 and 9. Two 3 nucleotide-insertion changes at ∼2.3 Mb on chromosome 2 in v5.3 genome assembly are found in *imp3* (Table 3). Since both changes are over 4 Mb away from the 2-98 and 55-193 markers, they were eliminated from further study. Two changes are found on chromosome 9 in v5.3 genome assembly (Table 3) and they are both within the mapping region of ∼8 cM defined by the SCA8-2 marker. Both changes map to chromosome 2 in the v4 genome assembly. The first SNP change, which maps to position 4,049,338 on chromosome 9, has 5 Illumina sequencing reads and a Phred quality score of 16.9. It is a G to A change that causes a non-synonymous change from R (cGg) to Q (cAg) in a RegA/RlsA-like protein (g9750). The exact same change is observed in an aflagellate mutant *cnc1* not included in the SNP/indel library (Dutcher and Nauman, unpublished) and thus is unlikely to be the causative change in the *imp3* mutant strain. The second change, which maps to position 3,721,280 on chromosome 9, has 122 reads and a Phred quality score of 214. It is a deletion of 9 nucleotides immediately before the stop codon of a PP2A catalytic protein (PP2A3, g9684) and the deletion is predicted to remove the last 3 amino acids, YFL, which are conserved in almost all type 2A phosphatases (PP2A, PP4, and PP6; Figure 3B, S1, and S2). This change was confirmed by Sanger sequencing.

### The PP2A catalytic subunits in *Chlamydomonas*

A previous study using sequence similarity indicated that the *Chlamydomonas* genome contains four potential PP2A catalytic subunits, PP2A-1c (g4366), PP2A3 (g9684), PP2A-c4 (Cre12.g494900), and PPA1 (Cre06.g308350) [34]. Due to the sequence similarities observed among PP2A, PP4, and PP6 in all organisms [32], we asked whether the four *Chlamydomonas* proteins are PP2A catalytic subunits using phylogenetic analysis. A phylogenetic tree was built based on 55 PP2A, PP4, and PP6 protein sequences from green algae (*Chlamydomonas reinhardtii*, *Chlorella variabilis*, *Micromonas*, *Ostreococcus lucimarinus*, *Ostreococcus tauri*, and *Volvox carteri*), yeast (*Saccharomyces cerevisiae*), land plants (*Arabidopsis thaliana* and *Zea mays*), invertebrates (*Caenorhabditis elegans* and *Drosophila melanogaster*), and mammals (*Mus musculus* and *Homo sapiens*) (Table S2, Figure 3A and S1). This phylogenetic tree shows that *Chlamydomonas* PP2A-1c and PP2A3 are PP2A-like proteins. PP2A-c4 is a PP4-like protein and PPA1 belongs to the PP6 family. Within the PP2A family, four subgroups are distinguished using a bootstrap analysis (Figure 3A). PP2A proteins from invertebrates, mammals, and a green alga *Chlorella* form subgroup 1 in the PP2A family. The subgroup 2 is composed of PP2A proteins from land plants and yeast. PP2A proteins from land plants and green algae are found in subgroup 3. The subgroup 4, which includes PP2A3, is a green algal-specific subgroup. Protein sequences of all 26 proteins from the PP2A family were aligned. The alignment reveals that while subgroups 1, 2, and 3 have the conserved T_304_PDYFL_309_ C-terminus, the proteins in the fourth subgroup do not have the conserved T_304_, instead, it is replaced with V, C, or M (Figure 3B and S2). This suggests that T_304_ is not conserved in the green algal-specific subgroup.

### Introduction of PP2A3 transgene into *imp3* rescues the mutant phenotype

**Figure 4:**
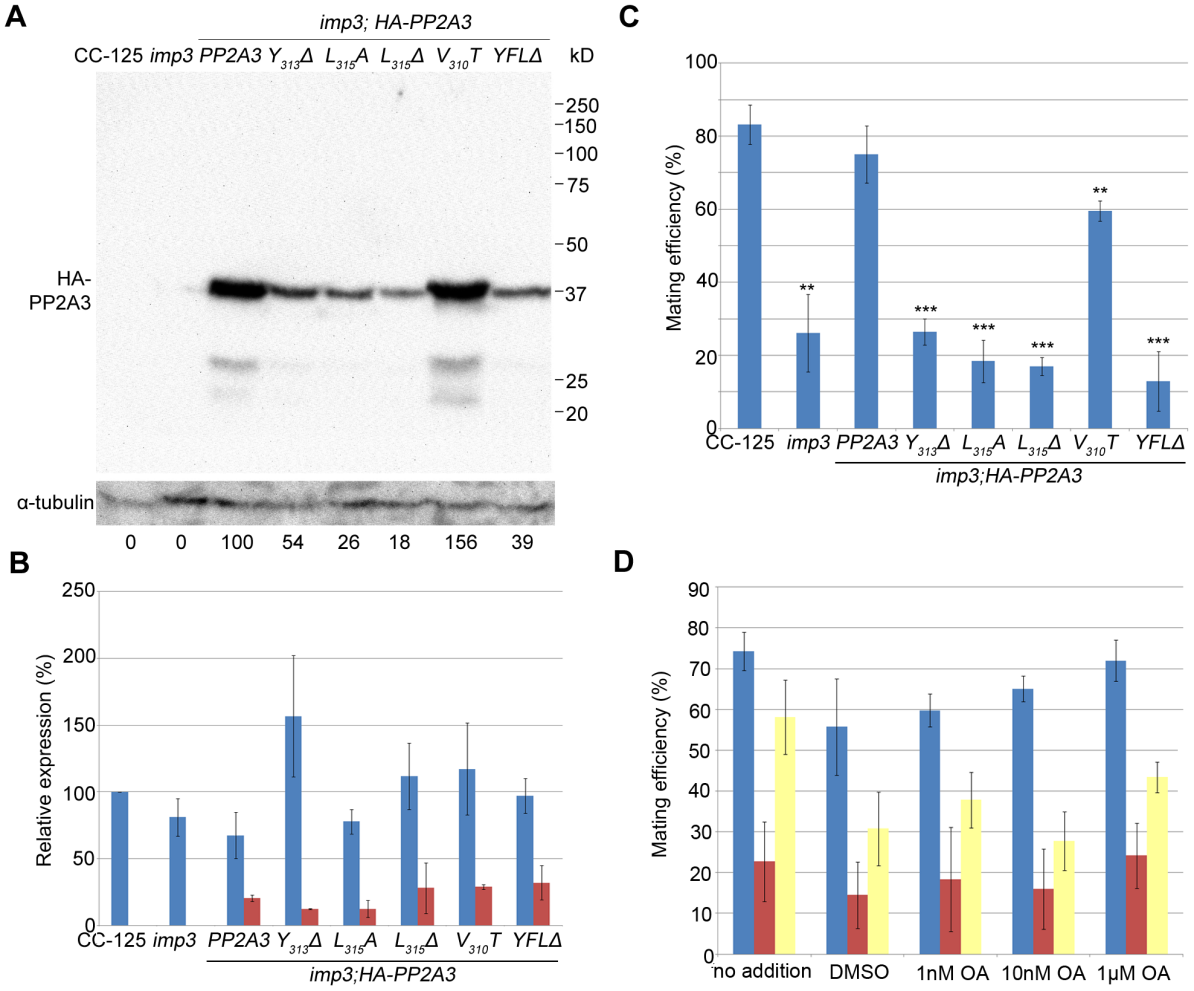
Introduction of HA-tagged PP2A3 transgene rescues the *imp3* mutant phenotype. (A) A representative immunoblot with the anti-HA antibody in transformants carrying either the wild-type or mutant *HA-PP2A3*. The HA-PP2A3 band is observed ∼37 kD and the two small bands (∼27 kD and ∼23 kD) may represent proteolyzed or truncated HA-PP2A3. The same membrane was re-probed with a monoclonal antibody against α-tubulin to serve as a measure of loading. Relative amount of the HA-PP2A3 protein in individual samples were measured by ImageJ and standardized by the amount of α-tubulin in the same sample. (B) Real-time PCR shows relative expression levels of *PP2A3* (blue) and *HA-PP2A3* (red) with the transcript level in wild-type CC-125 set at 100%. Each column represents three biological replicates and error bars show standard errors. (C) Mating efficiency of individual strains with the wild-type CC-124 after 1 hour of mating. Results represent three biological replicates and error bars show standard errors. **, p<0.01; ***, p<0.001. (D) Effects of okadaic acid on mating in wild-type (blue), *imp3* (red), and *imp3; HA-PP2A3* (yellow). The addition of DMSO serves as a control.

In order to demonstrate that the deletion of YFL at the C-terminus of PP2A3 is the causative mutation in *imp3*, we performed plasmid rescue. The *PP2A3* gene contains only a single exon, which is predicted to encode a 315 amino acid protein with a predicted molecular weight of 35,676 daltons. An HA-tagged *PP2A3* gene with the epitope tag HA immediately following the start codon to avoid compromising the C-terminus and under the regulation of the 637 bp endogenous *PP2A3* promoter was transformed into the *imp3* mutant strain, and whole cell extract from 6 putative transformants were screened by immunoblotting with an anti-HA antibody (Table S3). The HA-PP2A3 protein is predicted to have a molecular weight of 36,760 daltons. The anti-HA antibody recognized a ∼37 kD band in one transformant (*imp3; HA-PP2A3*, Figure 4A). Given the importance of Y_313_ and L_315_ (the equivalent of Y_307_ and L_309_ in mammalian cells) for the function of the PP2A catalytic subunit [33], we generated various N-terminal HA-tagged mutant forms (Y_313_Δ, L_315_A, L_315_Δ, and Y_313_F_314_L_315_Δ) under the same promoter and transformed them into the *imp3* cells individually (Table S3). All mutant forms of the HA-PP2A3 protein are expressed, as detected by the anti-HA antibody but the amount of the protein is significantly less than the transformed wild-type HA-PP2A3 protein (Figure 4A). Additionally, we generated a V_310_T substitution to investigate whether this change has any effect on rescue of the *imp3* mutant strain. The abundance of this protein is about 1.5 fold higher than the wild-type *HA-PP2A3* transformant (Figure 4A). An immunoblot with a monoclonal antibody against α-tubulin was used to quantify protein loading (Figure 4A). Two smaller bands (∼27 kD and ∼23 kD) were also detected by the anti-HA antibody and they may correspond to proteolyzed/truncated PP2A3.

To ask whether the difference in protein abundance observed in the *HA-PP2A3* transformants is due to the abundance of the transgenic *HA-PP2A3* transcript or due to protein stability, we measured the transcript levels of *PP2A3* by real-time PCR. In the wild-type strain CC-125 and *imp3*, real-time PCR detected only transcript levels of the endogenous *PP2A3* transcript (Figure 4B, blue). In all *HA-PP2A3* transformants, levels of two transcripts were detected. The first primer set detected the combined transcript levels of endogenous *PP2A3* and transgenic *HA-PP2A3* (Figure 4B, blue). Overall transcript levels of *PP2A3* in all strains tested are comparable. The second primer set detected only the transcript level of transgenic *HA-PP2A3* (Figure 4B, red). The transcript levels of transgenic *HA-PP2A3* in all transformants are about one-quarter of the total *PP2A3* transcript levels. There is no significant difference among different mutant transformants compared to the wild-type *HA-PP2A3* transformant (Figure 4B, red). Therefore, we conclude the differences observed in the protein levels of HA-PP2A3 in different mutant transformants are not due to the abundance of the transgenic *HA-PP2A3* transcripts, but rather due to the stability of the HA-PP2A3 proteins.

Transformation of wild-type *HA*-*PP2A3* into *imp3* cells successfully rescued the mating defect (Figure 4C, *HA-PP2A3*). Mating between *imp3; HA-PP2A3 plus* and *minus* gametes and mating between wild-type and *imp3; HA-PP2A3* both produce thick pellicles (Figure 2A). The V_310_T change, which is found in 5 independent transformants, partially rescues the mating efficiency to about 60% (Figure 4C). None of the changes in the YFL motif rescues the mating phenotype (Figure 4B, Y_313_Δ, L_315_A, L_315_Δ, and YFLΔ), which indicates the importance of the last 3 amino acids in the function of PP2A3 during mating. Thus, we conclude that PP2A3 is encoded by the *IMP3* gene and the deletion of nine nucleotides at the C-terminus of this gene causes the defective mating efficiency of *imp3* cells.

We further asked whether inhibition of PP2A3 has any effect on mating efficiency. Okadaic acid (OA), a polyether fatty acid, was shown to inhibit the phosphatase activity of PP2A [40], enhance phosphorylation of Y_307_ [41], and inhibit methylation of L_309_ [42], [43] *in vitro*. OA inhibits PP2A at very low concentrations and the dissociation constant (*Ki*) between OA and PP2A is ∼0.032 nM [44]. From *in vivo* studies, however, the amount of OA required to inhibit PP2A varies from 10 nM in human lung cancer cells [45] to ∼1 µM in MCF7 breast cancer cells [46]. It is suggested that the entry rate of OA can be affected by pH, temperature, and exposure time to OA [47]. We tested the effect of OA on *Chlamydomonas* mating at concentrations of 1 nM, 10 nM, and 1 µM. The mating efficiency between wild-type CC-124 (*mt−*) and CC-125 (*mt+*) is around 75% (Figure 4D). The addition of DMSO and different concentration of OA, for one hour at room temperature, has no significant effect on the mating efficiency (Figure 4D, blue bars). Similarly, addition of DMSO or OA has no significant effect on the mating efficiency of *imp3 mt+*×*imp3 mt−* (Figure 4D, red bars) and *imp3; HA-PP2A3 mt+*×*imp3; HA-PP2A3 mt−* (Figure 4D, yellow bars). Pre-treatment of cells with autolysin, an enzyme that removes *Chlamydomonas* cell walls, before the addition of OA, leads to similar results (data not shown). In a study on phosphoproteome in *Chlamydomonas*, cells pre-incubated with 1.5 µM OA for 29 hours accumulate 38% more phosphorylated proteins [48]. Therefore, our OA results indicate that either one hour inoculation is not sufficient for OA to enter *Chlamydomonas*, or the effect of OA on *Chlamydomonas* is more complicated than simple inhibition of PP2A3.

### PP2A3 interacts with a PP2A scaffold protein and localizes to both cell bodies and flagella

**Figure 5:**
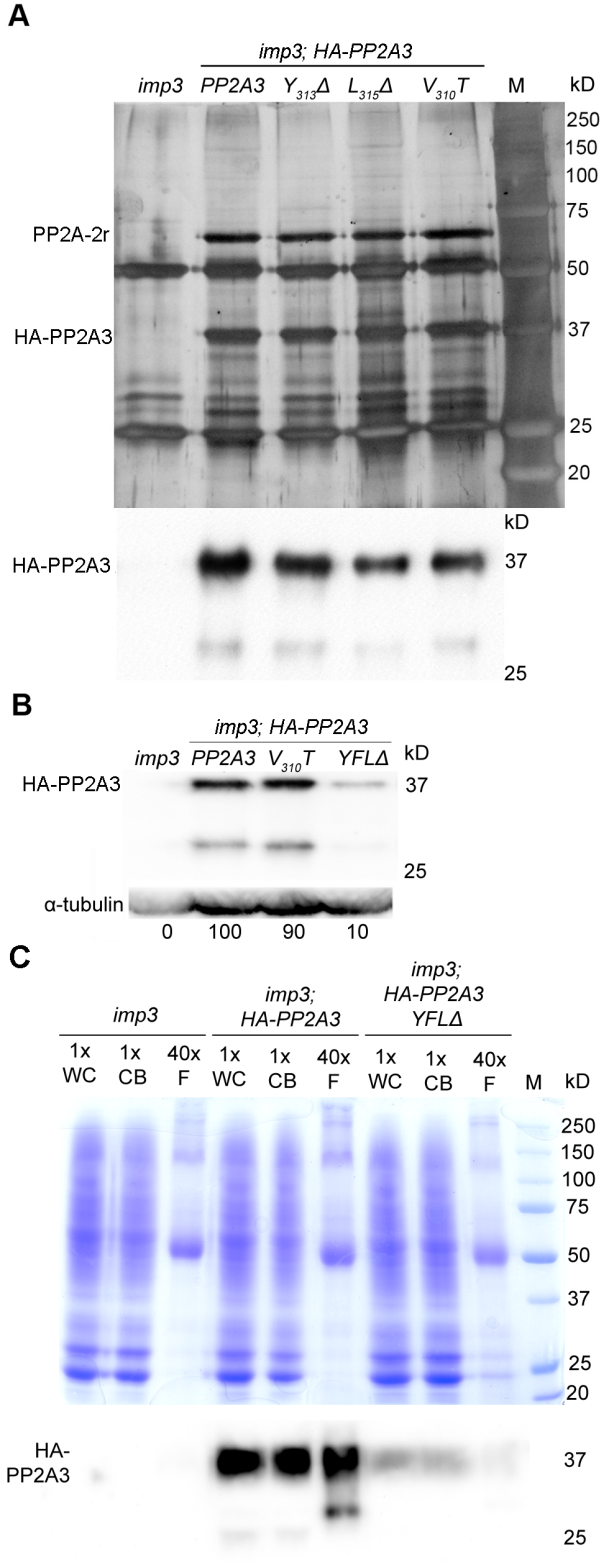
HA-PP2A3 interacts with a scaffold subunit protein and localizes to the flagella. (A) Upper panel, immunoprecipitation of wild-type and mutant HA-PP2A3 proteins from whole cell extracts of gametes by anti-HA-antibody. Proteins were separated on a 10% polyacrylamide gel and visualized by silver staining. M, protein standards. Bottom panel, immunoblot with the anti-HA antibody shows a band with the expected size (∼37 kD) and a faint band with a smaller size (∼29 kD). (B) A representative immunoblot with the anti-HA antibody to detect HA-PP2A3 from flagellar proteins in *HA-PP2A3* transformants. The same membrane was re-probed with a monoclonal antibody against α-tubulin to serve as a measure of loading. Relative amount of the HA-PP2A3 protein in individual samples were measured by ImageJ and standardized by the amount of α-tubulin in the same sample. (C) Relative distribution of HA-PP2A3 in whole cells (WC), cell bodies (CB), and flagella (F). Protein from about 1.25×10^7^ whole cells and cell bodies, and flagella isolated from about 5×10^8^ cells were used. Upper panel, Coomassie blue staining of a 10% polyacrylamide gel to visual protein extracts from different compartments of cells. Lower panel, immunoblot with the anti-HA antibody.

To identify interacting proteins of PP2A3 and to investigate whether changes in the YFL motif lead to changes in protein-protein interactions, we performed immunoprecipitation with the anti-HA antibody. Two major bands of ∼65 kD and ∼37 kD are obtained by immunoprecipitation from whole cell extract from *imp3* gametes transformed with wild-type *HA-PP2A3* but not with untransformed *imp3* gametes (Figure 5A). The ∼37 kD band is the HA-PP2A3, indicated by an immunoblot probed with an anti-HA antibody (Figure 5A). The ∼65 kD band was excised and subjected to mass spectrometry. The protein with the most number of peptides (94; 27 are unique) is PP2A-2r (Cre11.g477300) and it has a predicted size of 64,729 daltons. Changes in *Y_313_Δ, L_315_Δ*, *V_310_T*, or *YFLΔ* did not affect the pull-down of PP2A-2r by the HA antibody (Figure 5A and S3). Mass spectrometry of the ∼65 kD band pulled down by HA-PP2A3-*V_310_T* and by HA-PP2A3-*YFLΔ* resulted in 68 (27 unique) and 82 (26 unique) peptides of PP2A-2r, respectively. Thus, the interaction between the catalytic subunit PP2A3 and the scaffold subunit PP2A-2r is not affected by the changes at the C-terminus of PP2A3. PP2A-2r is one of the two PP2A scaffold proteins in the genome and was found in the flagellar proteome [34], [49]. Another scaffold subunit FAP14, which is also found in the flagellar proteome, has a predicted molecular weight of 100,787 daltons. Given that no significant band at ∼100 kD was identified in the immunoprecipitation (Figure 5A and S3), it is unlikely that PP2A3 interacts with FAP14.

Since PP2A-2r is present in the flagellar proteome, we asked whether PP2A3 localizes to the flagella. Flagella were isolated from *imp3* and the HA-tagged transformants with the wild-type *PP2A3, V_310_T, and YFLΔ* genes. The HA-PP2A3 is detected in both wild-type and mutant transformants, but not in the untransformed *imp3* flagella with the anti-HA antibody (Figure 5B). The *YFLΔ* transgene strain contains only ∼10% of HA-PP2A3 of those found in the wild-type *HA-PP2A3* and the *V_310_T* strain; this is similar to observations in the whole cell extract immunoblots (Figure 4A). A smaller band (∼29 kD), which may represent proteolyzed/truncated HA-PP2A3, is again recognized by the anti-HA antibody (Figure 5B).

To compare the relative distribution of HA-PP2A3 in *Chlamydomonas* cell bodies and flagella, we perform immunoblots of HA-PP2A3 on the basis of cell equivalents (Figure 5C). Protein from equal numbers of whole cells and cell bodies, and from flagella isolated from about 40 times more cells, were used in the analysis. In both *imp3; HA-PP2A3* and *imp3; HA-PP2A3 YFLΔ* strains, the HA-PP2A3 signal intensity is comparable in all three portions (Figure 5C). While the flagellar proteins represent less than 5% of protein found in whole cell extract [50], we do not find a significant enrichment of HA-PP2A3 in the flagella. In contrast, we observed a significant reduction of the HA-PP2A3 signal in *imp3; HA-PP2A3 YFLΔ* when compared to *imp3; HA-PP2A3*. Similar to previous observation (Figure 4A and Figure 5B), we noticed additional smaller bands in whole cells, cell bodies, and flagella. It is intriguing that the smaller bands observed in whole cells/cell bodies and in flagella are different in size and intensity. It is likely that these represent truncated PP2A3 proteins but the functions of these truncated proteins are unknown.

### Localization of PP2A3 changes in the *imp3* mutant strain and in mating cells

**Figure 6:**
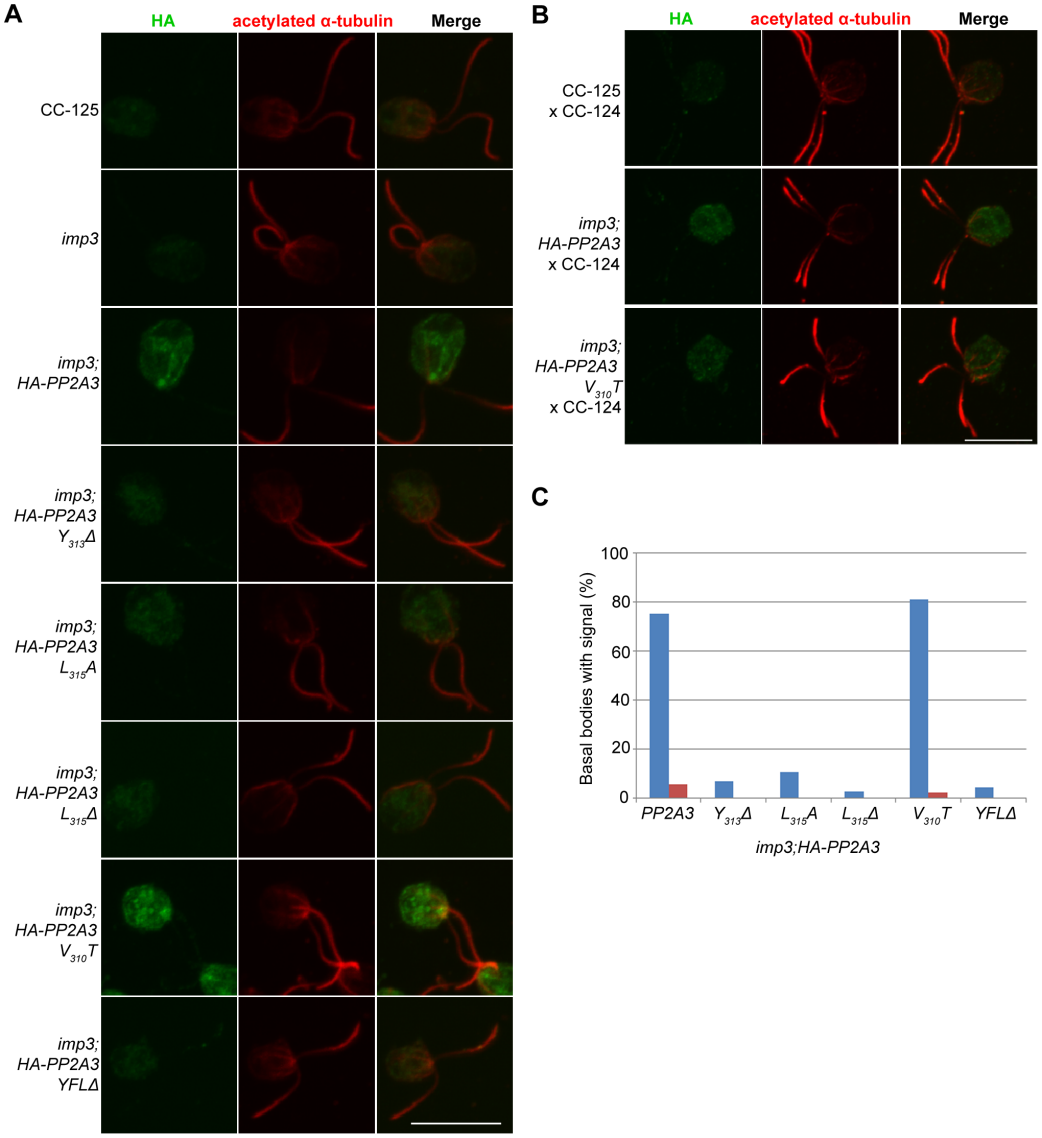
HA-PP2A3 localizes to the basal body region in gametes. (A) Immunofluorescence with the anti-HA antibody (green, first column) in unmated gametes of wild-type (CC-125), *imp3*, or *imp3* transformed with wild-type and mutant forms of *HA-PP2A3*. Immunofluorescence with the anti-acetylated α-tubulin (red, second column) identifies the *Chlamydomonas* flagella. Merged images of both staining are shown in the third column. Scale bar, 10 µm. (B) Immunofluorescence of the HA-PP2A3 location in wild-type dikaryons (CC-125×CC-124), in dikaryons between wild-type and *imp3* gametes transformed with wild-type or *V_310_T HA-PP2A3*. (C) Percentages of cells in each strain that showed the basal body localization of the HA-PP2A3 signal. Blue columns represent results from the unmated gametes. Red columns represent data from dikaryons identified by their four flagella. For each point, over 50 cells were collected.

To ask where the HA-PP2A3 protein localize, we performed immunofluorescence with the HA antibody in six transformant strains (Figure 6A). In wild-type (CC-125) and untransformed *imp3* gametes, there is some non-specific binding of the antibody in the cell body. In *imp3; HA-PP2A3* gametes, robust signals are observed throughout the cells. In addition, in ∼80% of *imp3; HA-PP2A3* gametes, accumulation of the signal is observed around the basal body area (Figure 6C, blue bars). The same signal intensity and localization is observed in *imp3* cells transformed with *HA-PP2A3* carrying a V_310_T mutation. In comparison, in *imp3* cells transformed with the mutant forms of *HA-PP2A3* (*Y_313_Δ*, *L_315_A*, *L_315_Δ*, and *YFLΔ*), the signal intensities of HA are significantly reduced, consistent with what we observed in the immunoblots (Figure 4A). Less than 10% of these cells showed basal body localization (Figure 6C). Therefore, we conclude that mutations of the terminal YFL affect the localization of PP2A3 to the basal body region.

Given the accumulation of the HA-PP2A3 proteins in wild-type *HA-PP2A3* and *V_310_T* cells, we asked whether mating of these gametes with wild-type gametes would lead to change of localization of HA-PP2A3 (Figure 6B). When *Chlamydomonas* cells mate, the flagella adhere to each other, leading to cell fusion to form a single cell with four flagella and two nuclei, which is known as a dikaryon. We examined dikaryons one hour after mixing wild-type gametes (CC-125×CC-124); they show a low background of non-specific staining. In contrast, dikaryons formed between *imp3; HA-PP2A3* (wild-type or *V_310_T*) and wild-type gametes show strong signals throughout the cells. However, less than 5% of these dikaryons show staining around the basal body area (Figure 6C, red bars). These results indicate that PP2A3 moves out of the basal body region in dikaryons.

## Discussion

### Whole genome sequencing to generate a *Chlamydomonas* SNP/indel library

Whole genome sequencing has become an important tool to allow quick identification of causative mutations in *Chlamydomonas* ([18] and Dutcher *et al.*, submitted), *Caenorhabditis elegans* [16], *Drosophila* [51], and humans [52]. In *Drosophila*, direct comparison of sequences from parental and EMS mutagenized chromosomes leads to the identification of causative SNPs. This removes the need for sequence alignment to the reference genome sequences, which eliminates the natural variation of SNPs within different strains [51]. However, this approach is not feasible to identify mutants whose original strain backgrounds are unavailable. In *C. elegans*, a cross to a highly polymorphic strain and whole genome sequencing of a pool of 50 F2 progeny eliminates the need for meiotic mapping. The number of SNPs drops significantly within a ∼2 Mb region where the mutation resides. Thus, the number of SNPs of interest is reduced dramatically; it becomes easier to identify the causative mutation [16]. In the studies of human variants, databases such as dbSNP and The 1000 Genomes Project [53] are available to filter non-causative SNPs/indels. The filtering results in a reduction of ∼98% of SNPs/indels in a given individual and thus it becomes feasible to identify causative mutations for rare Mendelian diseases [52]. Similar to the human 1000 Genomes Project, a 1001 Genomes Project on *Arabidopsis thaliana* was initiated in 2008 [19]. Sequencing of 80 *Arabidopsis* strains identified ∼5.7 million SNPs/indels [54].

We previously used meiotic mapping to narrow the regions of interest to 269 kb in NG6/*fla8-3* and 458 kb in NG30/*ift80*, respectively. Identification of one and six nonsynonymous changes in these regions eventually led to discovery of the causative mutations in these mutant strains [18]. In an approach similar to that used in *C. elegans* [16], we combined a pool of 14 progeny from a cross between a *pf27* mutant strain and the highly polymorphic S1C5 strain for whole genome sequencing. This approach narrows the region of interest to ∼2 Mb on chromosome 12 ([55] and Alford *et al.*, submitted), and eliminates the need for genome-wide mapping. However, the number of SNPs within 2 Mb remains large and it is hard to identify the causative mutation easily without further fine scale mapping. Therefore, generation of a SNP/indel library similar to the databases generated in other organisms is necessary to further eliminate common SNPs/indels found in *Chlamydomonas* strains.

While the two major laboratory strains (CC-124 and CC-125) were isolated from a single diploid zygote, 137c, they have >80,000 SNP/indel difference (Table 1). Both strains, and the reference strain used for genome assembly, belong to Subline III, as described by Pröschold *et al.* [39]. Therefore, strains that belong to Subline I, and strains from Subline II, such as *uni1*, are expected to contain more changes. The two isolo strains, originated from CC-124 and CC-125, after 10 rounds of backcrosses to CC-124, are expected to contain changes similar to each other and to CC-124. Instead, in addition to the expected difference observed on chromosome 6, the isolo strains show >30,000 polymorphisms between each other on chromosomes 3 and 17 (Figure 1A and Table 2). It is worth noted that the sequencing of isolo strains was performed on the Genome Analyzer IIx platform with 36 base-pair single-end reads and the sequencing of almost all other strains except *uni1* was performed on the HiSeq platform with 101 base-pair paired-end reads. Thus, the quality of alignments and coverage on the genome are slightly lower in the isolo strain sequence. As a result, SNP/indel calling in the isolo strains may be less comprehensive than that in other strains (Figure 1A, Table 1, and Table 2). Nevertheless, the accumulation of changes on chromosomes 3, 6, 12, 16, and 17 found in CC-124 is observed in one or both isolo strains. The difference observed between isoloP and isolo M on chromosomes 3 and 17 suggests that in addition to the recombination-suppressed regions found on chromosome 6, there are additional regions on these two chromosomes that may show suppression of meiotic recombination. It is unclear why these regions are recombination-suppressed.

Given that many *Chlamydomonas* mutants were generated from the 137c background (Table S4), the SNP/indel library that we generated greatly facilitates quick identification of causative mutations in six mutant strains, *fla18, fla24, fla9* (Dutcher *et al.*, submitted), *imp3* (this study), *pf7*, and *pf8* (Dutcher *et al.*, manuscript in preparation). The percentage of unique SNPs/indels after filtering is about 1∼2% in most strains and 7% in *imp3* (Table 1). Even though we cannot eliminate the need for meiotic mapping in this study, we were able to combine this SNP/indel library and transcriptional profiles during flagellar regeneration [56] to identify mutations in flagellar assembly genes without the need of meiotic mapping (Dutcher *et al.*, submitted). Thus, we anticipate that this SNP/indel library will facilitate rapid discovery of more causative mutations in *Chlamydomonas* mutant strains.

### The importance of the terminal YFL in *Chlamydomonas* PP2A3

In humans, there are two isoforms (α and β) of PP2A catalytic subunits (PP2Ac). They share high sequence identity (97%), but the α isoform is ∼10 times more abundant than the β isoform at both transcript and protein levels [31]. In the invertebrates, *Drosophila* [57] and *C. elegans* [58], only one PP2A is present in each genome. In contrast, *Arabidopsis* has at least five PP2Ac proteins, and they can be divided into two subfamilies based on sequence similarity. The sequence identity between two subfamilies, I and II, is ∼80% [59]. The subfamily I proteins are involved in abscisic acid (ABA) and brassinosteroid signaling [60], [61]. The subfamily II proteins play an important role in auxin distribution and plant development [59]. In green algae, there are two PP2Ac proteins present in the genome. While one *Chlamydomonas* PP2A protein (PP2A-1c) belongs to the same group as the *Arabidopsis* PP2A subfamily I, the other *Chlamydomonas* PP2A protein, PP2A3/IMP3, is found in a green algal-specific cluster (Figure 3A). The sequence identity between PP2A-1c and PP2A3 is only 63%. While our results suggest that PP2A3 functions in flagella, it is likely that PP2A3 and its homologs in the green algae-specific cluster have additional functions since *Ostreococcus* lacks flagella.

Our study on PP2A3 provides several insights in the importance of the terminal YFL in PP2A. First, it is necessary for PP2A function in *Chlamydomonas* mating. Transgenes with mutations of individual amino acids or deletion of all three amino acids fail to rescue the mating defect (Figure 4C). Second, mutations or deletion of YFL do not affect the binding of PP2A3 to the scaffold subunit. In a study on the structure of the PP2A holoenzyme *in vitro*, the removal of the last 15 amino acids at the C-terminus of human PP2A does not affect the formation of the holoenzyme, which includes the catalytic subunit, the scaffold subunit, and the regulatory subunit PR61 γ1 isoform [62]. In cultured mammalian cell lines including COS7, NIH 3T3, and neuro-2a, changes of T_304_, Y_307_ and L_309_ alter the binding between PP2A and different regulatory subunits [63], [64]. Our study provides evidence that YFL is not required to form the core PP2A enzyme, which contains the catalytic C subunit and the scaffold A subunit (Figure 5A and S3), but it is unclear whether it can form the holoenzyme since the regulatory subunit that interacts with PP2A3 has not been identified. Third, loss of YFL affects the protein stability of PP2A3 in *Chlamydomonas*. It is not clear if this instability is observed in all kinds of cells. In mammalian cell lines, deletion or mutation of T_304_, Y_307_ and L_309_ do not affect the stability of HA-tagged PP2Ac as judged by immunoblots probed with an anti-HA antibody [63], [64]. However in yeast cells, contradictory results were obtained with tagged versus untagged proteins [65], [66]. It suggests that the HA tag might destabilize the PPH21p-L_369_Δ mutant [66]. In our study, the change or deletion of Y_313_, L_315_, and deletion of YFL, but not the V_310_T change, leads to reduction of PP2A3 protein levels. We think it is unlikely that the HA tag has an effect on the stability of PP2A3 mutants, but to completely rule out the possibility, a PP2A3-specific antibody will be necessary. Fourth, loss of YFL affects the localization of PP2A3 to the basal body region in *Chlamydomonas*. In *C. elegans* embryo, LET-92, the catalytic subunit of PP2A, localizes to centrosomes as well as in the cytoplasm. The centrosome localization of LET-92 depends on RSA-1 (Regulator of Spindle Assembly 1), a B″ type PP2A regulatory subunit that also localizes to centrosomes [67]. In monkey kidney CV-1 cells, immunofluorescence using a monoclonal antibody against PP2Ac revealed that PP2Ac localizes to both microtubule and centrosomes [68]. It was shown in HeLa cells both the B′ type PP2A regulatory subunit B56α and the PP2A scaffold subunit localize to the centrosomes and it was proposed that B56α serves as a chaperone that facilitates the translocation and function of the catalytic subunit [69]. In *Chlamydomonas*, the basal bodies serve as nucleation sites for assembly of flagella during interphase and function as centrioles during mitosis [70]. Our finding that PP2A3 localize to the basal body region is consistent with PP2Ac being found in centrosomes of other organisms. More importantly, our result suggests that mutations in the terminal YFL affect the basal body localization of PP2A3. It is likely that PP2A3 binds to a *Chlamydomonas* regulatory subunit similar to RSA-1 or B56α in the centrosomes and mutations in PP2A3 YFL attenuate the interaction between PP2A3 and the regulatory subunit.

### PP2A3 and *Chlamydomonas* mating

In the *Chlamydomonas* mating signaling pathway triggered by agglutination of flagella from *plus* and *minus* cells, two proteins have identified. The TRPP2 protein Polycystin-2 (PKD2; Cre17.g715300) localizes to the flagellar membrane as well as to the cell body and is enriched fourfold in flagella during gametogenesis [71]. A cGMP-dependent protein kinase (PKG/CGK2; Cre02.g076900) is also present in both the cell body and flagella. Flagellar agglutination during mating leads to phosphorylation of a tyrosine residue in PKG that activates its protein kinase activity [50]. Knockdown of *PKD2* and *PKG* via RNA interference leads to reduced mating efficiency, which resembles the *imp3* phenotype [50], [71]. The signal transduction cascade is postulated to involve at least one additional uncharacterized tyrosine kinase that phosphorylates PKG [50].

Given the localization of PP2A3 to the basal body region as well as to the flagella, we propose that PP2A3 regulates the mating signaling pathway through one or more of the following mechanisms. Similar to other signaling pathways, phosphorylation and dephosphorylation of components in the mating signaling pathway need fine regulation. As a phosphatase, PP2A3 may act as a negative regulator of phosphorylation of PKG. The presence of PP2A3 keeps PKG unphosphorylated in gametes. In the *imp3* gametes, since the amount of the PP2A3 protein is significantly reduced, dephosphorylation of PKG is likely to be compromised. Alternatively, PP2A3 could act as a positive regulator to facilitate the movement of signaling proteins as well as agglutinin proteins into the flagella to allow the proper functions of these proteins on the flagellar membrane during mating. It has been shown that the transport of PKD2 to the flagellar membrane requires intraflagellar transport (IFT) [71] but the entry of a truncated SAG1 protein to the flagellar membrane is IFT-independent [72]. It is possible that PP2A3 acts either on these proteins directly or on the transport machineries themselves. Comparison of PKD2 levels in wild-type and *imp3* cells, as well as exploration of the relationship between PP2A3 and IFT will provide some answers to the function of PP2A3 in protein trafficking to the flagellar membrane.

The involvement of important signaling proteins such as PKD2 makes the mating process of *Chlamydomonas* an outstanding model to study ciliary/flagellar signaling. Our finding that PP2A3 involved in this process suggests that this ubiquitous enzyme may play an important role in this signaling. In addition, identification of the causative mutation in the *imp4* mutant strain, which shares the same phenotype as *imp3* but is unlinked to *imp3* [21], may reveal additional players in this intriguing pathway.

## Materials and Methods

### *Chlamydomonas* strains

*Chlamydomonas reinhardtii* strains, CC-124 (mt−), CC-125 (mt+), CC-1952 (S1C5), CC-3864 (*fla18*), CC-3866 (*fla24*), CC-1918 (*fla9*), CC-2668 (*ida3*), CC-465 (*imp3*), CC-3660 (*pf23*), CC-568 (*pf7*), CC-560 (*pf8*), CC-1926 (*uni1*), CC-916 (NG30, *ift80*), were obtained from the *Chlamydomonas* Resource Center. Both isoloM (CC-4403, mt−) and isoloP (CC-4402, mt+) were generated in this laboratory and deposited to the *Chlamydomonas* Resource Center. All mutant strains obtained were backcrossed at least once with either CC-124 or CC-125, and progeny with the mutant phenotype were used in further analysis. The *cnk10* mutant strain was generated by insertional mutagenesis in the CC-125 background.

### Whole genome sequencing and read alignment

*Chlamydomonas* genomic DNA was prepared as previously described [18]. Approximately 10^8^ cells were used in DNA preparation. About three µg of genomic DNA from each strain was submitted to Genome Technology Access Core (Department of Genetics, Washington University in St. Louis) for Illumina sequencing. The 36 bp single-ended (SE) sequencing of isoloP and isoloM and the 60 bp pair-ended (PE) sequencing of *uni1* were performed with the Genome Analyzer IIx platform. The 101 bp PE sequencing of the other 13 strains was performed with the HiSeq platform. Almost all samples with the exception of *ift80* and *uni1* were individually tagged with a unique 7-nucleotide index and multiple samples were subjected to one sequencing flow cell lane (Table S4). The sequencing reads of *ift80* were obtained from a previously published result [18]. The indexed sequencing reads were de-multiplexed before being subjected to read alignment.

For read alignment, the genome sequence of *Chlamydomonas* v5.3.1 (Creinhardtii_236.fa.gz) was downloaded from http://www.phytozome.net/chlamy.php [36]. An indexed database was built from 54 FASTA sequences (17 chromosomes+37 scaffolds) by Novoindex (novocraft.com). The SE sequencing reads were aligned to the database by Novoalign (version 2.08.02) with the following options: -o SAM -r random -l 25 -e 100 -a AGATCGGAAGAGCGGTTCAGCAGGAATGCCGAG -H -c 12. The PE sequencing reads were aligned to the database by Novoalign with the following options: -o SAM -r random -l 30 -e 100 -i 230 140 -a AGATCGGAAGAGCGGTTCAGCAGGAATGCCGAG AGATCGGAAGAGCGTCGTGTAGGGAAAGAGTGTA -H -c 12 -h 90 120. SAMtools (version 0.1.18) [73] was used to convert the resulting SAM (Sequence Alignment/Map) files to BAM (Binary Sequence Alignment/Map) format and sort the BAM files. Duplicated reads were removed by Picard (version 1.46; picard.sourceforge.net) and the resulting BAM files were sorted by SAMtools.

### SNP calling and the SNP/indel library

The same genomic DNA sequence (v5.3.1) was indexed by SAMtools and used as a reference file in SAMtools mpileup (options: -u -g), which outputs a BCF (Binary Call Format) file for BCFtools (options: -b -v -c -g; vcfutils.pl varFilter -D 999) to call for SNP/indel variants using Bayesian inference [73]. The numbers of SNPs/indels from individual strains were obtained from the resulting VCF (Variant Call Format) files.

VCF files of all 16 strains sequenced are available on the SNP/indel library webpage (http://stormo.wustl.edu/SNPlibrary/). The webpage is designed to allow direct comparison of SNPs/indels from one strain to one or more strains of interest. It also allows users to upload their own VCF files and compare to SNPs/indels from one or more of the 16 strains we provide. The output is a VCF file which maintains its original format minus the common SNPs/indels. To detect unique changes in the *imp3* mutant strain, we compared the SNPs/indels to the SNPs/indels in all other 15 strains. The resulting new VCF file was then subjected to SnpEff (version 3.0j) [74] to identify SNPs/indels within the coding regions or exon/intron boundaries (options: -no-upstream -no-downstream -no-intergenic -no-intron -no-utr -hom -o txt). The SnpEff reference database for was built based on the genome assembly and gene annotation (Creinhardtii_236_gene.gff3.gz) of Chlamydomonas v5.3.1 [36].

### Meiotic mapping of *imp3*

In order to quickly identify the *imp3* progeny from crosses between *imp3* and wild-type cells as well as between *imp3* and S1C5 cells, we developed a low-density mating assay. In this assay, approximately 1o^6^ cells were subjected to 0.1 ml of nitrogen-free medium (M-N/5) in a well of a 96-well plate for 4 hours at 21°C. Half of the volume of cells was mixed with *imp3 mt+* testers and half were mixed with *imp3 mt−* testers. Starting at 30 minutes after mixing and at 30-minute increments for four hours, the cells were monitored under the dissecting microscope for agglutination and the formation of pellicle. The cells were incubated under constant light overnight at 21°C and then scored again after 18 hours. Mating between *IMP3* cells and *imp3* testers formed multi-layer, dark green pellicle around the four-hour time point, which was maintained until the 18-hour time point. Mating between *imp3* and *imp3* testers formed a thin, single-cell layer beginning around the two-hour time point but it never accumulated into the multi-layer pellicle within the next 16 hours. In the cross between *imp3* and S1C5, over 100 independent *imp3* progeny were picked from over 100 tetrads and used for meiotic mapping.

Crude DNA was obtained from about 10^6^ cells from each progeny. Cells were resuspended in 10 µl 1× Vent Buffer (20 mM Tris-HCl, 10 mM (NH_4_)_2_SO_4_, 10 mM KCl, 2 mM MgSO_4_, 0.1% Triton X-100, pH 8.8) and 1 mg/ml proteinase K (Sigma-Aldrich). The cells were incubated at 58°C for 30 minutes for proteinase K activity and 95°C for 15 minutes to inactivate proteinase K. For PCR, 0.5 µl of crude DNA was used in 20 µl reactions. Each PCR sample contained 1× TAQ buffer (50 mM KCl, 10 mM Tris-HCL, 0.1% Triton X-100, pH 9.0), 1.5 mM MgCl2, 0.4 µM dCAPS primers, 0.4 mM dNTPs, 5% DMSO and 4% TAQ polymerase. The cycling protocol was 95°C for 2 minutes, and 30 cycles of 95°C for 20 seconds, annealing temperature for 20 seconds (Table S1), and 72°C for 1 minute, followed by a final extension time of 5 minutes at 72°C. If digestion of the PCR product was necessary to detect the polymorphism, 10 µL of the PCR product, 1.2 µL of an enzyme's corresponding buffer, 1.2 µL of 10× BSA if necessary, 0.2 µL of the enzyme, and the remaining volume with water to reach a final 15 µL. This was placed at 37°C from 5 minutes to 1 hour depending on the enzyme's efficiency as suggested by the manufacturer (New England Biolabs).

### Protein sequence alignment and phylogenetic tree

The protein sequence of PP2A3 was used in BLAST on NCBI and 55 proteins with the expected E-value less than or equal to 1E-100 were collected from green algae (*Chlamydomonas reinhardtii*, *Chlorella variabilis*, *Micromonas*, *Ostreococcus lucimarinus*, *Ostreococcus tauri*, and *Volvox carteri*), yeast (*Saccharomyces cerevisiae*), land plants (*Arabidopsis thaliana* and *Zea mays*), invertebrates (*Caenorhabditis elegans* and *Drosophila melanogaster*), and mammals (*Mus musculus* and *Homo sapiens*) (Table S2). Sequences alignment was performed with MUSCLE [75] and the output ClustalW (strict) file was subjected to Colorfy [76] for color-coded protein sequence alignment.

From the protein sequence alignment (Figure S1), the low similarity region (∼30 amino acids from the N-terminus) was trimmed from individual protein sequences (Table S2) and multi-sequence alignment was again performed by MUSCLE. The output Phylip interleaved file was subjected to SEQBOOT for 100 bootstraps in the PHYLIP package (3.69; http://evolution.genetics.washington.edu/phylip.html). The 100 sets of samples were subjected to PROTDIST, which uses protein distance matrix to calculate distance. An unrooted tree was generated by a neighbor-joining method implemented by NEIGHBOR for each set of samples. A consensus tree from the 100 unrooted trees was computed by CONSENSE. The output tree was visualized by DRAWGRAM and the bootstrap numbers for each branch were obtained from the outfile file generated by CONSENSE (Figure 3A).

Based on the phylogenetic tree, proteins from the PP2A subfamily were collected and subjected to another round of multi-sequence alignment by MUSCLE and color-coded by Colorfy (Figure S2). Sequence alignment of amino acids close to the C-terminus is shown in Figure 3B.

### Plasmid DNA constructs and *Chlamydomonas* transformation

The full-length *PP2A3* genomic DNA, which contains 637 bp upstream of the start codon, the 948 bp coding region, and 424 bp downstream of the stop codon was amplified by primers PP2A-inF2 and PP2A-inR (Table S5) with the Phusion DNA polymerase (New England Biolabs) with the following cycling condition: 98°C for 30 sec, and 30 cycles of 98°C for 10 seconds, 69°C for 10 seconds, and 72°C for 1 minute, followed by a final extension time of 2 minutes at 72°C. The resultant PCR product was gel-purified by UltraClean 15 DNA Purification Kit (MO BIO Laboratories) and cloned into a pBlueScript vector digested with *EcoR*V using the In-Fusion HD Cloning Kit (Clontech). The resultant *PP2A3*-pBS plasmid was subjected to Sanger sequencing (GENEWIZ) to ensure the sequence accuracy.

The HA tag was inserted right after the start ATG codon via nested PCR. DNA polymerase and cycling conditions were the same as above with the exception of extension time was reduced to 20 seconds. The primers (PP2A-HA-F, PP2A-3R, Table S5) in the first round of PCR with the *PP2A3*-pBS plasmid generated above as a template, introduced the HA tag and generated a 361 bp fragment, which was gel purified and used as a template in the second round of PCR. A second forward primer (PP2A-3F-HA), along with the same reverse primer (PP2A-3R), produced a 399 bp fragment. This fragment was then digested with *Avr*II and *Afl*II, gel purified, and cloned into *PP2A3*-pBS digested with the same enzymes to generate the *HA-PP2A3*-pBS plasmid. The plasmid was subject to Sanger sequencing (GENEWIZ) to ensure the sequence accuracy.

The mutations to the C-terminus of the protein were introduced by knitting PCR. DNA polymerase and cycling conditions were the same as above with the exception of extension time was 30 seconds. Using the *HA-PP2A3-*pBS plasmid as a template, the PP2A3-inf-3F primer was paired with different reverse primers each bearing a mutation (Y313Δ-R; Y315A-R; L315Δ-R; V310T-R; YFLΔ-R, Table S5) to amplify ∼430 bp bands. The PP2A3-inf-3R primer was paired with different forward primers each bearing a mutation (Y313Δ-F; Y315A-F; L315Δ-F; V310T-F; YFLΔ-F, Table S5) to amplify ∼410 bp bands. All bands were gel purified and the corresponding bands (i.e., PP2A3-inf-3F-Y313Δ-R and Y313Δ-F-PP2A3-inf-3R) were used as templates in the second round of PCR, with PP2A3-inf-3F and PP2A3-inf-3R as primers. The resultant ∼830 bp products from the second round of PCR, were gel purified, digested with *Nco*I and *Pml*I and cloned into *HA-PP2A3*-pBS plasmid digested with the same enzymes. All mutant plasmids were subject to Sanger sequencing (GENEWIZ) to ensure they bear the correct mutations.

*Chlamydomonas* transformation was performed as previously described with modification [76]. One µg of plasmid DNA was used in each transformation into the *imp3* cells, using 1 µg of the pSI103 plasmid, which confers resistance to paromomycin [77], for co-transformation. Paromomycin-resistant (paro^R^) cells were selected on TAP plates with 10 µg/ml paromomycin. In these TAP plates, the concentration of glacial acetic acid was 0.75 ml/L instead of 1 ml/L [35]. The reduction of acetic acid in the medium confers more robust paromomycin resistance. Crude DNA was prepared from paro^R^ colonies and PCR with primers PP2A-HA-short and PP2A-4R (Table S5) was performed to identify HA-positive colonies. The HA-positive colonies were then subjected to immunoblots with anti-HA antibody to confirm the presence of HA-tag in the transformants. The number of paro^R^ colonies, number of HA-positive colonies by PCR and by immunoblot are summarized in Table S3.

### Immunoblots and immunofluorescence

The primary antibodies used in this study were anti-HA High Affinity antibody (3F10, Roche, 1∶2000 dilution in immunoblots and 1∶200 dilution in immunofluorescence), anti-α-tubulin antibody (Sigma-Aldrich, T6199, 1∶2000 dilution in immnoblots), and anti-acetylated- α-tubulin antibody (Sigma-Aldrich, T7451, 1∶250 dilution in immunofluorescence). The secondary antibodies used for immunoblots were HRP-conjugated goat anti-mouse antibody (BioRad, 1∶5000), and HRP-conjugated goat anti-rat antibody (Sigma-Aldrich, 1∶5000). Alexa 488-conjugated goat anti-rat antibody (Invitrogen, 1∶500) and Alexa 594-conjugated goat anti-mouse antibody (Invitrogen, 1∶500) were used in immunofluorescence.

Cells used in immunoblots were gametes, which were cultured on R plates for 5 days at 25°C and resuspended in nitrogen-free medium (M-N/5) for 4 hours at room temperature prior to protein extraction. For whole cell extracts, ∼10^7^ cells were resuspended in 30 µl of 20 mM HEPES (pH 7.0) solution with the addition of 1× ProteaseArrest (G-Biosciences) and 10% SDS. The samples were incubated at 37°C for 5 minutes and diluted with 90 µl of 20 mM HEPES (pH 7.0) and 1× ProteaseArrest [78]. For flagellar extracts, ∼5×10^9^ *Chlamydomonas* cells were resuspended in 4°C HEPES/Sr/DTT buffer (pH 7.1) buffer. Flagella were then detached from cells by pH shock on ice, collected by centrifugation at 4°C [79], and resuspended in 40 µl of 20 mM HEPES (pH 7.0) and 1× ProteaseArrest.

Protein extract from all samples isolated were store at −80°C. The amount of protein in each sample was measured by Bradford assay (Bio-Rad protein assay) before loaded into 10% polyacrylamide gels. The proteins were transferred to PVDF membrane (Millipore) after SDS-PAGE, blocked with 5% nonfat dry milk in PBST (Phosphate Buffered Saline with 0.02% Tween-20) at room temperature for 1 hour, probed with primary antibody in 3% milk in PBST overnight at 4°C, washed with PBST 3 times, 5 minutes each, probed with secondary antibody in 3% nonfat dry milk in PBST 1 hour at room temperature, and washed with PBST 3 times, 5 minutes each. ECL Plus Western Blotting Detection Reagents (GE Healthcare Life Sciences) or SuperSignal West Femto Chemiluminescent Substrate (Thermo Scientific) was used to expose the signal, which was captured by a FluorChem H2 image (Alpha Innotech). Signal quantification analysis was performed by ImageJ (NIH).

For immunofluorescence, gametes were treated with autolysin for 30 minutes before fixed with 2% paraformaldehyde as previously described [80]. The autolysin treatment was omitted in immunofluorescence of the dikaryons. The images were captured with an UltraVIEW VoX laser scanning disk confocal microscope (PerkinElmer) and acquired by Volocity software (PerkinElmer).

### Immunoprecipitation and mass spectrometry

Immunoprecipitation was performed as described by Olson *et al.* [81] with modifications. About 10^9^ gametes were used and proteins were cross-linked with freshly prepared DSP (Dithiobis-succinimidyl propionate; Pierce) on ice for 30 minutes before the cross-link was stopped by the addition of 100 mM Tris-HCl (pH 7.5). Cells were lysed by sonication and lysates were centrifuged at 20,000 g for 30 minutes at 4°C to remove insoluble materials. Protein concentration from the supernatant was determined by Bradford assay. One microgram of anti-HA high affinity antibody (3G10) was inoculated with 20 µl of protein G magnetic beads (Invitrogen) for 30 minutes at room temperature and cross-linked with 5 mM BS^3^ (Bis[sulfosuccinimidyl] suberate; Pierce) in conjugation buffer (20 mM sodium phosphate, pH 7.0, 0.15 M NaCl) for 30 minutes at room temperature. The cross-link reaction was stopped by the addition of 50 mM Tris-HCl (pH 7.5) and incubated at room temperature for 15 minutes. The anti-HA-antibody-conjugated protein G magnetic beads were then incubated with cell lysates that contain ∼12 mg of proteins in each sample overnight at 4°C. The beads were washed and the bound proteins were eluted with 1× SDS PAGE loading buffer with the addition of 100 mM DTT at 90°C for 10 minutes. About 1/10 of the elution was used in SDS-PAGE and immunoblots with anti-HA antibody and the rest was separated by a 10% polyacrylamide gels and stained by either silver [82] or 0.1% Coomassie Brilliant blue R-250. The ∼65 kD band stained by Coomassie blue was cut out and sent to the Taplin Mass Spectrometry Facility (Harvard University) for mass spectrometry.

### Real-time RT-PCR

*Chlamydomonas* total RNA extraction and real-time RT-PCR was performed as previously described [76]. Primers used in these reactions were PP2A3-HA-short and PP2A3-4R for *HA-PP2A3* transcripts and PP2A3-7F and PP2A3-3R for endogenous+*HA-PP2A3* transcripts (Table S5).

## Supporting Information

Figure S1 Sequence alignment of 55 protein phosphatases from 13 organisms. Sequence similarity percentage is displayed by colors shown below the alignment. Organism abbreviation: At, *Arabidopsis thaliana*; Ce, *Caenorhabditis elegans*; Cr, *Chlamydomonas reinhardtii*; Cv, *Chlorella variabilis*; Dm, *Drosophila melanogaster*; Hs, *Homo sapiens*; M, *Micromonas sp. RCC299*; Mm, *Mus musculus*; Ol, *Ostreococcus lucimarinus*; Ot, *Ostreococcus tauri*; Sc, *Saccharomyces cerevisiae*; Vc, *Volvox carteri*; Zm, *Zea mays*.
(TIF)

Figure S2 Sequence alignment of 26 protein phosphatas 2A proteins from 13 organisms. Sequence similarity percentage is displayed by colors shown below the alignment. Organism abbreviation: At, *Arabidopsis thaliana*; Ce, *Caenorhabditis elegans*; Cr, *Chlamydomonas reinhardtii*; Cv, *Chlorella variabilis*; Dm, *Drosophila melanogaster*; Hs, *Homo sapiens*; M, *Micromonas sp. RCC299*; Mm, *Mus musculus*; Ol, *Ostreococcus lucimarinus*; Ot, *Ostreococcus tauri*; Sc, *Saccharomyces cerevisiae*; Vc, *Volvox carteri*; Zm, *Zea mays*.
(TIF)

Figure S3 Immunoprecipitation of HA-PP2A3. Upper panel, immunoprecipitaton of wild-type and mutant HA-PP2A3 proteins from whole cell extract by anti-HA-antibody. Proteins were separated on a 10% polyacrylamide gel and visualized by Coomassie blue staining. M, protein standards. Bottom panel, immunoblot with the anti-HA antibody shows a band with the expected size (∼37 kD) and a faint band with a smaller size (∼27 kD).
(TIF)

Table S1 Markers used to map *imp3*.
(DOCX)

Table S2 Protein phosophatases used to build a PP2A-PP4-PP6 phylogenetic tree.
(DOCX)

Table S3 Summary of HA-PP2A3 transformants screening.
(DOCX)

Table S4 Sequence indexes used in different strains.
(DOCX)

Table S5 Primers used in this study.
(DOCX)
