# Supplementary figures and images for: Whole Genome Sequencing Identifies a Deletion in Protein Phosphatase 2A That Affects Its Stability and Localization in Chlamydomonas reinhardtii
Source: PLoS Genet. 2013 Sep 26;9(9):e1003841. doi: 10.1371/journal.pgen.1003841 (PMC3784568; doi:10.1371/journal.pgen.1003841)

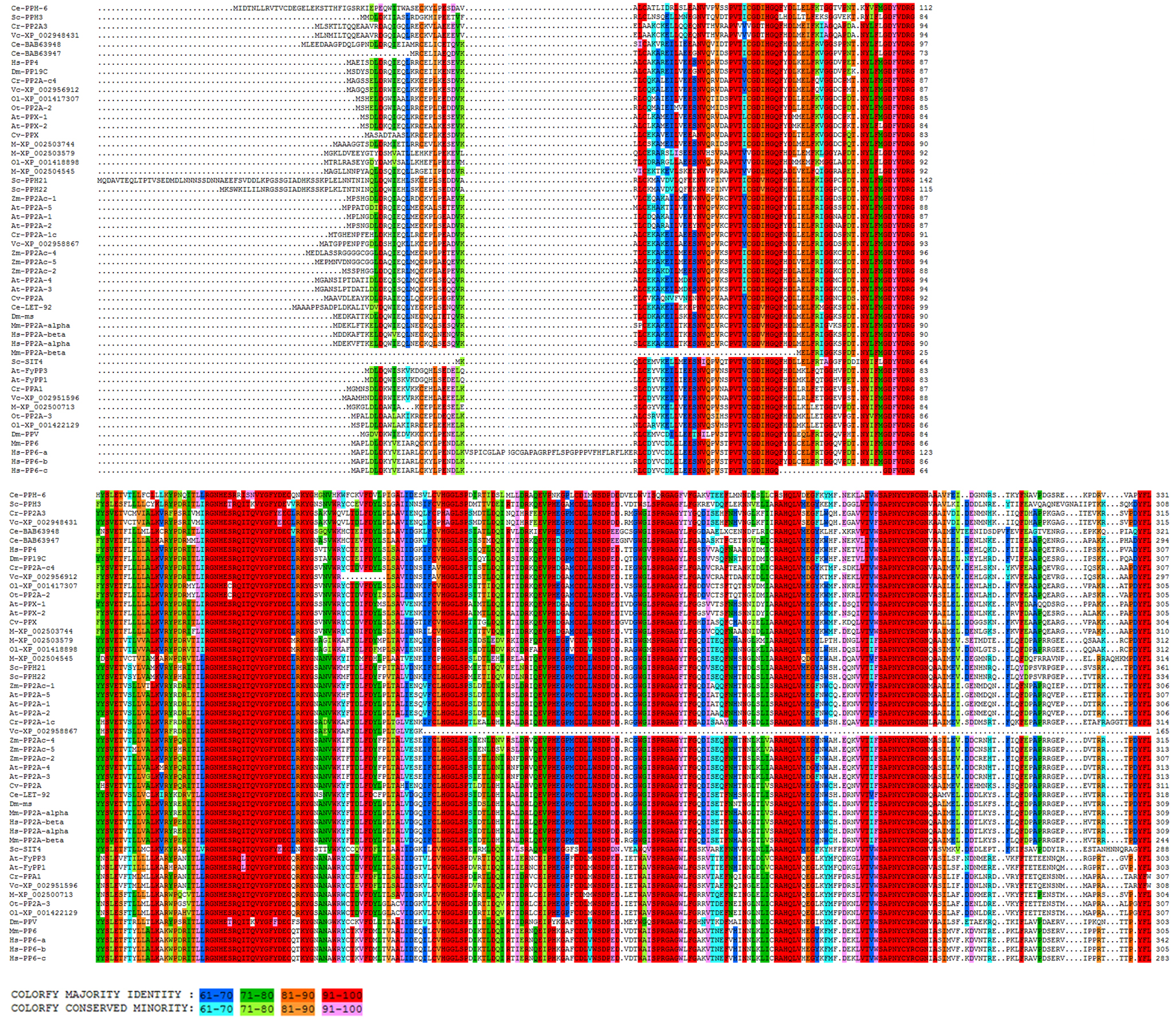

Supplement: Figure S1 — Sequence alignment of 55 protein phosphatases from 13 organisms. Sequence similarity percentage is displayed by colors shown below the alignment. Organism abbreviation: At, Arabidopsis thaliana; Ce, Caenorhabditis elegans; Cr, Chlamydomonas reinhardtii; Cv, Chlorella variabilis; Dm, Drosophila melanogaster; Hs, Homo sapiens; M, Micromonas sp. RCC299; Mm, Mus musculus; Ol, Ostreococcus lucimarinus; Ot, Ostreococcus tauri; Sc, Saccharomyces cerevisiae; Vc, Volvox carteri; Zm, Zea mays. (TIF) [file pgen.1003841.s001.tif]

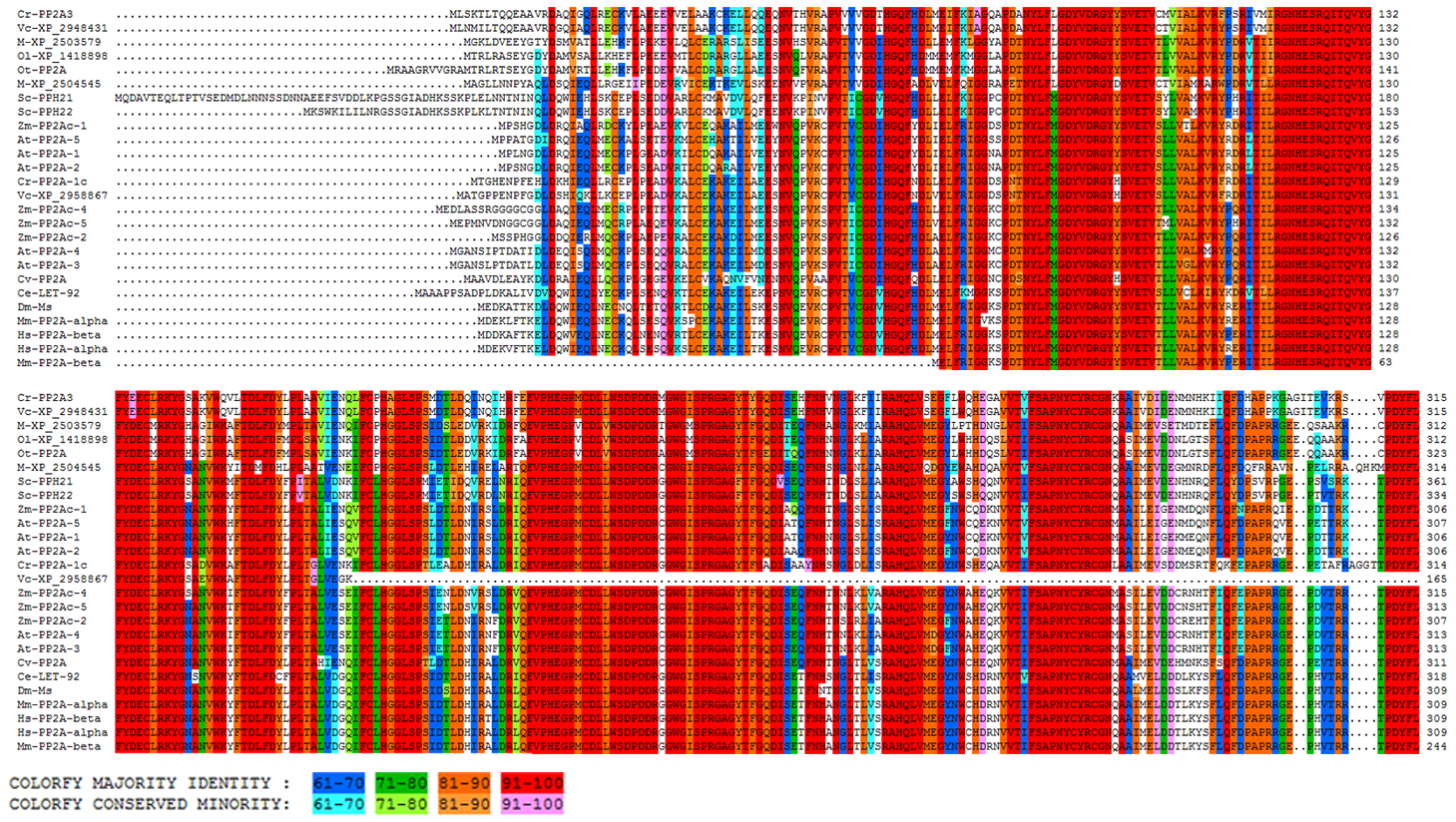

Supplement: Figure S2 — Sequence alignment of 26 protein phosphatas 2A proteins from 13 organisms. Sequence similarity percentage is displayed by colors shown below the alignment. Organism abbreviation: At, Arabidopsis thaliana; Ce, Caenorhabditis elegans; Cr, Chlamydomonas reinhardtii; Cv, Chlorella variabilis; Dm, Drosophila melanogaster; Hs, Homo sapiens; M, Micromonas sp. RCC299; Mm, Mus musculus; Ol, Ostreococcus lucimarinus; Ot, Ostreococcus tauri; Sc, Saccharomyces cerevisiae; Vc, Volvox carteri; Zm, Zea mays. (TIF) [file pgen.1003841.s002.tif]

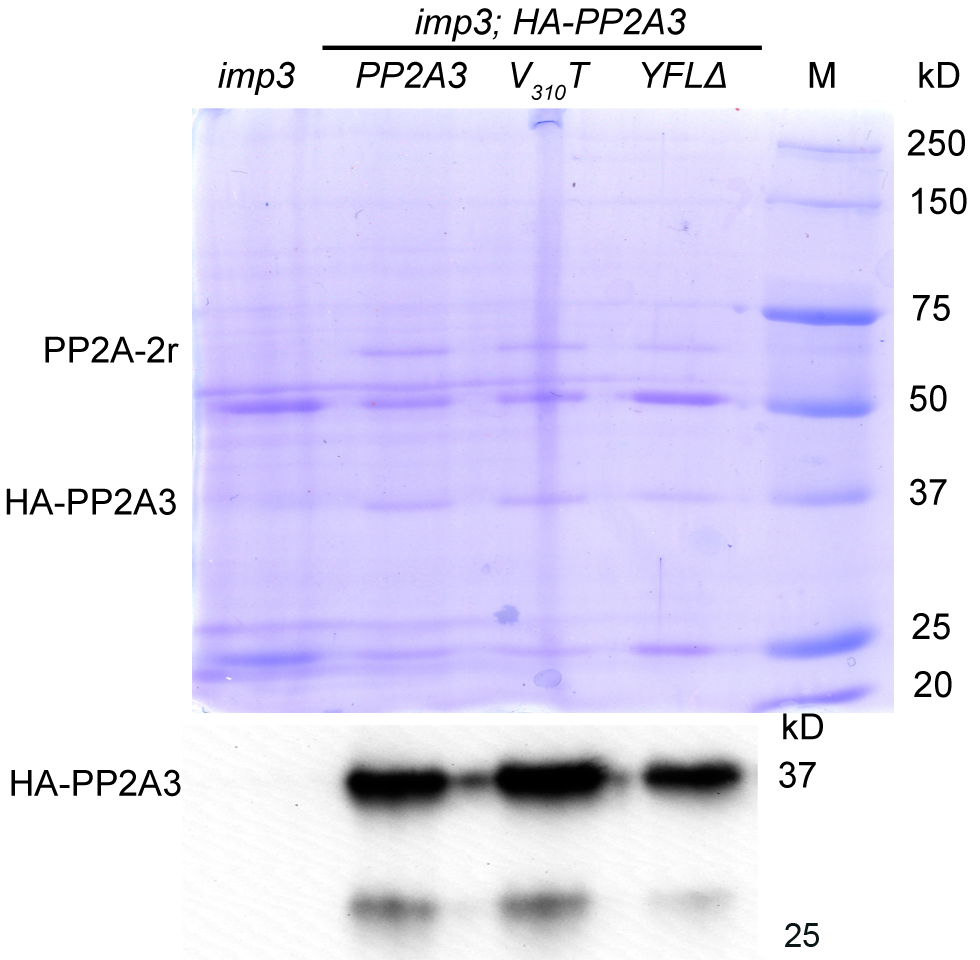

Supplement: Figure S3 — Immunoprecipitation of HA-PP2A3. Upper panel, immunoprecipitaton of wild-type and mutant HA-PP2A3 proteins from whole cell extract by anti-HA-antibody. Proteins were separated on a 10% polyacrylamide gel and visualized by Coomassie blue staining. M, protein standards. Bottom panel, immunoblot with the anti-HA antibody shows a band with the expected size (∼37 kD) and a faint band with a smaller size (∼27 kD). (TIF) [file pgen.1003841.s003.tif]
