# Supplementary material for: Whole Genome Sequencing Identifies a Deletion in Protein Phosphatase 2A That Affects Its Stability and Localization in Chlamydomonas reinhardtii
Source: PLoS Genet. 2013 Sep 26;9(9):e1003841. doi: 10.1371/journal.pgen.1003841 (PMC3784568; doi:10.1371/journal.pgen.1003841)
Supplement: Table S1 — Markers used to map imp3. (DOCX) [file pgen.1003841.s004.docx]

**Table S1. Markers used to map *imp3*.**

| **Chr^a^** | **Marker** | **P:R^b^** | **Primer 1 (P1)** | **Primer 2 (P2)** | **Temp** | **Enzyme** | **P1 start position^a^** | **P2 start position^a^** |
| --- | --- | --- | --- | --- | --- | --- | --- | --- |
| **1** | CNK | 9:9 | TGAGCAGGAGATCCTGGAGT | AGCGAAGTCTGTGTCGTCCT | 55 | ScrFI | 321657 | 322308 |
| **1** | SCA18-2 | 7:7 | GGGCGGAGTACCATAGGACGTAG | TGTATGAACCACACACGGTCACAC | 56 | EcoRI | 7680887 | 7680603 |
| **2** | CGK2 | 9:11 | ACTTCTGTCCGGTGGTACCTGCCTG | CAATATGTCACTGGCTACGGCAAG | 56 | PstI | 500084 | 499928 |
| **2** | GSP1 | 11:4 | GGGGTAGGCGGGTAGCAGGCCTGCA | CACAGCGCCTACCACAGTATTCCT | 58 | PstI | 5594189 | 5594351 |
| **2** | 2-98 | 104:22 | ACACGCATATTGCCGTTAACTCACT | TTGCGACAAAACACGCCTTATCTA | 54 | PstI | 6591734 | 6591564 |
| **2^c^** | 2-98 |  |  |  |  |  | 56858506^c^ | 5685658^c^ |
| **2** | 55-193 | 154:5 | ACCCCCGCTCTCCCTCAAACCTCGA | GTTTAGCAACTGTAGTCCGGGCAG | 58 | EcoRV | 7120181 | 7120347 |
| **2^c^** | 55-193 |  |  |  |  |  | 9839682^c^ | 9839515^c^ |
| **3** | SCA67 | 12:7 | GCTTGAGGGCGGTTTGGAGAGT | AGCAATCGTGCGCATTCCTCAG | 58 | XbaI | 348185 | 348413 |
| **3** | TSB | 8:12 | AAGTCTACTGTTCGCATTGCTG | GTCCTCACGCTTTAGGTAAATCTC | 48 | AlwNI | 2714177 | 2713365 |
| **3** | DMAT | 9:11 | GGACATTCGTGTGGAGTGAA | GGGCACGTCTGACAGTAACA | 48 | - | 3734876 | 3734575 |
| **3** | TUA1 | 7:13 | GGCCATGAGTTGCTCTTTC | AGGAAACGCAGTCAAGGGTA | 53 | MspI | 6017805 | 6018305 |
| **3** | PHOT1 | 12:8 | TGCAGTTTTGCAGTTTGGAG | CTGCCGTCCATGTTCCTTAT | 51 | DdeI | 8322182 | 8321757 |
| **4** | TUA2 | 6:13 | CACAAACACAAGTACCGTTGGT | AAGGTCAGGGGGAAGATCC | 55 | HaeIII | 147181 | 146829 |
| **4** | PF20 | 4:16 | CATGAGCATCCAACGATAGGTA | CCCCATTTTGATGAAGAAGTTG | 55 | - | 3483812 | 3485382 |
| **5** | 51-610 | 9:11 | TGTCCGACATGGGTGGCGTGAAGCT | AGACAGAAGCATCTTGCCCAGG | 57 | HindIII | 375928 | 375765 |
| **5** | ALDO | 9:11 | GTTGGAGTTCCATCGTCGTT | GTGGCTCCTCTCTGAACTGC | 53 | - | 2060958 | 2060642 |
| **5** | 181-55 | 9:11 | TGAGCGGCGAAAGGTGCGGCGCGAA | CGCACCTCACTACGTCCGTACCAC | 61 | EcoRI | 1750265 | 1750359 |
| **6** | CDC48 | 10:10 | AGTCCGCAGCACTGGTCTAC | AAGCTTGCACTGCATTGTTG | 53 | MspI | 2650447 | 2650689 |
| **6** | PP1 | 12:7 | GCGACGATTATGTTTGCTGA | GTGTCACCCCGATTCAGTTT | 51 | MspI | 6432775 | 6432584 |
| **6** | 9-1152 | 6:10 | TCCAACGACTCGGCGCGTACCACAA | GCATCTTCGTGGGCTACCAGCA | 60 | HindIII | 8860504 | 8860606 |
| **7** | SCA44 | 11:9 | CATGTGGAGTGATATTTGTGAGCATG | CAGTTAGCAGTTGGCCCACGTCA | 57 | HindIII | 611518 | 611266 |
| **7** | FA4 | 11:8 | GGAACGGTGACTGTCGGTAG | ACGCAAAGCCGGTAACATAG | 53 | - | 779221 | 779523 |
| **7** | ZSP1 | 10:10 | GAGGTCGCGGGGGAA | GCGAGTGCGGAATCAAA | 53 | DdeI | 2064693 | 2065500 |
| **7** | FA2 | 8:12 | TCGTTGTGTGTGAGGTGGAT | CACGCAATGCATGAATAAGG | 50 | - | 5523966 | 5523739 |
| **8** | HSP70 | 7:11 | AGCTGCTGCAGGACTTCTTC | GCTGGTTGTCGGAGTAGGTC | 53 | HaeIII | 2520167 | 2519904 |
| **8** | SCA78 | 6:11 | TCTCTCACGCTCTCACACTCTTCC | GCGTTTGCTACCAGAACAAAAAGG | 55 | SalI | 3725231 | 3724947 |
| **9** | SCA8-2 | 114:10 | CTTAGAAGCTCGGTGGTGGCTTC | CTGCTAGCACTTTGCGGTGCTG | 58 | PstI | 3516261 | 3515976 |
| **2^c^** | SCA8-2 |  |  |  |  |  | 6604889^c^ | 6604626^c^ |
| **9** | TRX1 | 11:6 | TTCCGTCCTCACCAAAGTTC | CCAGCAGAATTGACAGCAAA | 51 | ScrFI | 2372870 | 2373076 |
| **10** | BLD10 | 3:7 | TGCTCATGTCGTTGTCGAACAAGCT | CCAAGAAGGGTGACCAGTTCAAG | 56 | HindIII | 107321 | 107404 |
| **10** | CNA83 | 9:11 | CGGCCGCTGAAGCTGCTGTGA | TGCACATACCTCTGACGCTCCACC | 59 | - | 3742160 | 3742420 |
| **10** | SCA83 | 2:9 | CAGTTGCACAAACCGACAAAGAAA | CTCGGTACACCCTGCCATTGCG | 55 | SalI | 5969941 | 59699663 |
| **11** | VFL2 | 14:5 | GGAGGAGCTCCAAGAGATGA | TCAAGATGGATAGGCCCAGT | 53 | MnlI | 2038008 | 2038298 |
| **11** | GP40 | 15:5 | CCCCGATTTCGAACTTGATA | ATAAGTGCGAGGGGGACACT | 51 | MspI | 3685100 | 3684724 |
| **12** | Sca109 | 7:11 | CAAACAACTAAGTCGGATTCCTTCTG | CGCAATGCTCAGGTACAGATGC | 53 | PstI | 7264687 | 7264964 |
| **12** | RPL9 | 9:10 | TAACTGACACGCTGGAATGC | GTGATAGCAAGGAGCCGAAG | 51 | HaeIII | 7484737 | 7484352 |
| **12** | SCA7-1 | 7:11 | CCAAGTGAGAGGACCGCAGTGG | GCTCTCATACAAGCAGCTCGGCTA | 58 | PstI | 9277873; 9288939 | 9278114; 9289180 |
| **13** | SCA21-272 | 9:11 | TACCCCCTTCCTTGAATCCACTGAC | TCCACAACACCATAGTTACCCCAAC | 56 | PstI | 2131221 | 2131012 |
| **13** | IGPS | 10:10 | ACTACGATGTCTGGCGAGGT | CGGGTCCATCTTCTTTGCTA | 53 | RsaI | 4491640 | 4492420 |
| **13** | IDA5 | 11:9 | AGTCCGAGTACGACGAGTCC | TGCAAGCTATCCACGTATGC | 53 | - | 4549911 | 4550205 |
| **14** | BE05 | 9:11 | CCGGTCAAGTAGGTGCTGTT | TACGTAGCGGGTTTGAGCTT | 55 | HaeIII | 1404729 | 1404503 |
| **14** | 1-2118 | 8:10 | CATACCCTGGCCCAACCCGCTCTGC | CGTTGACAATGATGTGCTGTTCTG | 55 | PstI | 2195589 | 2195693 |
| **14** | ZYS2 | 3:6 | ATCACGTAAGGAGGAGCAAG | ATTTGGAATCCTCTCGTCAA | 49 | RsaI | 3872055; 3889982 | 3872671; 3890568 |
| **16** | CYTC6 | 11:8 | AAGCGCGTTCATGGTTCGGCC | CATCACGCAAATGGACACGTTCCG | 56 | - | 1239988 | 1239778 |
| **16** | RAA2 | 7:5 | ATTGACCACTGCGGCGCTAGCG | TAGTAGGGGCATCCGTGGCTCTCG | 63 | - | 3132516 | 3131920 |
| **16** | SCA31 | 6:12 | GAGCCAGTGTGAGCCTTGTGTG | CAGTAAGGATGGGATGGGTGTGAG | 58 | SalI | 7253572; 7258253 | 7258035; 7253354 |
| **17** | 870001 | 8:12 | AGATGGCTAGCTGTCCTTGC | GTGACGCAGATGGTGTTGAC | 52 | MnlI | 451898 | 452198 |
| **17** | LC5 | 5:15 | ACGCAGATGCATGGTGTAAA | AAAGTCAATTGCACCCGGTA | 53 | MspI | 2324006 | 2324206 |
| **17** | FLA10 | 7:13 | CTGCGCGCCAGCAAGCTCAAGT | GGTAACAGCCCGTCTTCCAGGGCC | 63 | MspI | 4321530 | 4321040 |

1. Chromosomes and primer positions are based on Chlamydomonas genome assembly v5.3.
2. P:R, parental: recombinant; c. Indicates chromosomes and coordinates in *Chlamydomonas* genome assembly v4.
