# Supplementary material for: Whole Genome Sequencing Identifies a Deletion in Protein Phosphatase 2A That Affects Its Stability and Localization in Chlamydomonas reinhardtii
Source: PLoS Genet. 2013 Sep 26;9(9):e1003841. doi: 10.1371/journal.pgen.1003841 (PMC3784568; doi:10.1371/journal.pgen.1003841)
Supplement: Table S2 — Protein phosophatases used to build a PP2A-PP4-PP6 phylogenetic tree. (DOCX) [file pgen.1003841.s005.docx]

**Table S2. Protein phosophatases used to build a PP2A-PP4-PP6 phylogenetic tree.**

| **Protein** | **Accession #^a^** | **AA used^b^** | **Organisms** |
| --- | --- | --- | --- |
| **At-FyPP1** | NP_175454 | 24-303 | *Arabidopsis thaliana* |
| **At-FyPP3** | NP_188632 | 24-303 | *Arabidopsis thaliana* |
| **At-PP2A-1** | NP_176192 | 28-306 | *Arabidopsis thaliana* |
| **At-PP2A-2** | NP_172514 | 28-306 | *Arabidopsis thaliana* |
| **At-PP2A-3** | NP_567066 | 35-313 | *Arabidopsis thaliana* |
| **At-PP2A-4** | NP_565974 | 35-313 | *Arabidopsis thaliana* |
| **At-PP2A-5** | NP_177154 | 29-307 | *Arabidopsis thaliana* |
| **At-PPX-1** | NP_194402 | 25-305 | *Arabidopsis thaliana* |
| **At-PPX-2** | NP_200337 | 25-305 | *Arabidopsis thaliana* |
| **Ce-BAB63947** | BAB63947 | 14-294 | *Caenorhabditis elegans* |
| **Ce-BAB63948** | BAB63948 | 38-321 | *Caenorhabditis elegans* |
| **Ce-LET-92** | NP_502247 | 40-318 | *Caenorhabditis elegans* |
| **Ce-PPH-4.1** | NP_499603 | 53-333 | *Caenorhabditis elegans* |
| **Ce-PPH-4.2** | NP_001022898 | 38-321 | *Caenorhabditis elegans* |
| **Ce-PPH6** | NP_497714 | 53-331 | *Caenorhabditis elegans* |
| **Cr-PP2A-1c** | XP_001699424 | 32-314 | *Chlamydomonas reinhardtii* |
| **Cr-PP2A3^a^** | g9684^a^ | 35-315 | *Chlamydomonas reinhardtii* |
| **Cr-PP2A-c4** | XP_001700556 | 28-307 | *Chlamydomonas reinhardtii* |
| **Cr-PPA1** | XP_001691361 | 28-307 | *Chlamydomonas reinhardtii* |
| **Cv-PP2A** | EFN59957 | 33-311 | *Chlorella variabilis* |
| **Cv-PPX** | EFN51614 | 24-304 | *Chlorella variabilis* |
| **Dm-Microtubule star** | NP_476805 | 31-309 | *Drosophila melanogaster* |
| **Dm-PP19C** | NP_524803 | 28-307 | *Drosophila melanogaster* |
| **Dm-PPV** | NP_511061 | 25-303 | *Drosophila melanogaster* |
| **Hs-PP2A-alpha** | NP_002706 | 31-309 | *Homo sapiens* |
| **Hs-PP2A-beta** | AAV38333 | 31-309 | *Homo sapiens* |
| **Hs-PP4** | NP_002711 | 28-307 | *Homo sapiens* |
| **Hs-PP6-a** | NP_001116827 | 64-342 | *Homo sapiens* |
| **Hs-PP6-b** | NP_002712 | 27-305 | *Homo sapiens* |
| **Hs-PP6-c** | NP_001116841 | 27-283 | *Homo sapiens* |
| **M-XP_002500713** | XP_002500713 | 25-304 | *Micromonas sp. RCC299* |
| **M-XP_002503579** | XP_002503579 | 33-312 | *Micromonas sp. RCC299* |
| **M-XP_002503744** | XP_002503744 | 31-310 | *Micromonas sp. RCC299* |
| **M-XP_002504545** | XP_002504545 | 33-314 | *Micromonas sp. RCC299* |
| **Mm-PP2A-alpha** | AAD12587 | 31-309 | *Mus musculus* |
| **Mm-PP2A-beta** | EDL35413 | 1-244 | *Mus musculus* |
| **Mm-PP6** | NP_077171 | 27-305 | *Mus musculus* |
| **Ol-XP_001417307** | XP_001417307 | 26-305 | *Ostreococcus lucimarinus* |
| **Ol-XP_001418898** | XP_001418898 | 33-312 | *Ostreococcus lucimarinus* |
| **Ol-XP_001422129** | XP_001422129 | 27-307 | *Ostreococcus lucimarinus* |
| **Ot-PP2A** | XP_003080276 | 44-323 | *Ostreococcus tauri* |
| **Ot-PP2A-2** | XP_003078905 | 26-305 | *Ostreococcus tauri* |
| **Ot-PP2A-3** | XP_003084155 | 27-307 | *Ostreococcus tauri* |
| **Sc-PPH21** | EGA79648 | 83-361 | *Saccharomyces cerevisiae* |
| **Sc-PPH22** | EGA87691 | 56-334 | *Saccharomyces cerevisiae* |
| **Sc-PPH3** | NP_010360 | 25-308 | *Saccharomyces cerevisiae* |
| **Sc-SIT4** | EGA75745 | 4-288 | *Saccharomyces cerevisiae* |
| **Vc-XP_002948431** | XP_002948431 | 35-315 | *Volvox carteri* |
| **Vc-XP_002951596** | XP_002951596 | 29-308 | *Volvox carteri* |
| **Vc-XP_002956912** | XP_002956912 | 28-297 | *Volvox carteri* |
| **Vc-XP_002958867** | XP_002958867 | 34-165 | *Volvox carteri* |
| **Zm-PP2Ac-1** | ACG37926 | 28-306 | *Zea mays* |
| **Zm-PP2Ac-2** | ACG38746 | 29-307 | *Zea mays* |
| **Zm-PP2Ac-4** | NP_001150190 | 37-315 | *Zea mays* |
| **Zm-PP2Ac-5** | ACG34764 | 35-313 | *Zea mays* |

a. Accession numbers and protein sequences were obtained from NCBI with the exception of Cr-PP2A3. The protein sequence of Cr-PP2A3 (Accession number XP_001694950) is incomplete and thus we used the protein sequence from *Chlamydomonas* v5.3 genome assembly and the locus name of *Cr-PP2A3* is given instead.

b. Positions of amino acids used to build the phylogenetic tree shown in Figure 3A.
