# Supplementary material for: Whole Genome Sequencing Identifies a Deletion in Protein Phosphatase 2A That Affects Its Stability and Localization in Chlamydomonas reinhardtii
Source: PLoS Genet. 2013 Sep 26;9(9):e1003841. doi: 10.1371/journal.pgen.1003841 (PMC3784568; doi:10.1371/journal.pgen.1003841)
Supplement: Table S3 — Summary of HA-PP2A3 transformants screening. (DOCX) [file pgen.1003841.s006.docx]

**Table S3. Summary of HA-PP2A3 transformants screening.**

| **Plasmid** | **Paro^R^** | **HA PCR positive** | **Colonies used in immunoblot** | **HA immunoblot positive** |
| --- | --- | --- | --- | --- |
| ***HA-PP2A3*-pBS** | 20 | 6 | 6 | 1 |
| ***HA-PP2A3-Y_313_Δ*-pBS** | 96 | 37 | 10 | 1 |
| ***HA-PP2A3-L_315_A*-pBS** | 93 | 27 | 13 | 1 |
| ***HA-PP2A3-L_315_Δ*-pBS** | 96 | 36 | 36 | 1 |
| ***HA-PP2A3-V_310_T*-pBS** | 96 | 36 | 27 | 5 |
| ***HA-PP2A3-YFLΔ*-pBS** | 131 | 53 | 46 | 3 |
