# Supplementary material for: Whole Genome Sequencing Identifies a Deletion in Protein Phosphatase 2A That Affects Its Stability and Localization in Chlamydomonas reinhardtii
Source: PLoS Genet. 2013 Sep 26;9(9):e1003841. doi: 10.1371/journal.pgen.1003841 (PMC3784568; doi:10.1371/journal.pgen.1003841)
Supplement: Table S4 — Sequence indexes used in different strains. (DOCX) [file pgen.1003841.s007.docx]

**Table S4. Sequence indexes used in different strains.**

| **Strain** | **Index sequence** | **Number of samples per flow cell lane** | **Parental Strain** |
| --- | --- | --- | --- |
| **CC-124** | ATCGAGC | 6, with other samples | Zygote 137c |
| **CC-125** | ATGACAG | 4, with *fla18*, *fla24*, and *ida3* | Zygote 137c |
| **isolo M** | GCTTAGA | 2, with isolo P | CC-124 and CC-125 |
| **isolo P** | TGAGGTT | 2, with isolo M | CC-124 and CC-125 |
| **S1C5** | ATCGAGC | 4, with other samples | N/A |
| ***cnk10*** | TGAGGTT | 9, with other samples | CC-124 |
| ***fla18*** | TGAGGTT | 4, with CC-125, *fla24*, and *ida3* | CC-125 and crossed by S1C5 |
| ***fla24*** | GCTTAGA | 4, with CC-125, *fla18*, and *ida3* | CC-125 |
| ***fla9*** | GCTTAGA | 2, with another sample | CC-70; derived from 137c |
| ***ida3*** | CACCTCC | 4, with CC-125, *fla18*, and *fla24* | CC-124 or CC-125 |
| ***ift80*** | n/a | 1 | 137c |
| ***imp3*** | GCTTAGA | 4, with *pf23* and two other samples*.* Ran in 2 flow cells lane and sequencing reads were combined | CC-125 |
| ***pf23*** | ATGACAG | 4, with *imp3* and two other samples*.* Ran in 2 flow cells lane and sequencing reads were combined | 137c |
| ***pf7*** | TGAGGTT | 3, with *pf8* and another sample | 137c |
| ***pf8*** | GCTTAGA | 3, with *pf7* and another sample | 137c |
| ***uni1*** | n/a | 1 | CC-1009 or CC-1010 |
