# Supplementary material for: Whole Genome Sequencing Identifies a Deletion in Protein Phosphatase 2A That Affects Its Stability and Localization in Chlamydomonas reinhardtii
Source: PLoS Genet. 2013 Sep 26;9(9):e1003841. doi: 10.1371/journal.pgen.1003841 (PMC3784568; doi:10.1371/journal.pgen.1003841)
Supplement: Table S5 — Primers used in this study. (DOCX) [file pgen.1003841.s008.docx]

**Table S5. Primers used in this study**

| **Primer** | **Sequence** |
| --- | --- |
| **PP2A-inF2^a^** | CGGTATCGATAAGCTTGATGGCCTAAAGCGCCACATGCT |
| **PP2A-inR^a^** | CCGGGCTGCAGGAATTCGATCACGGCAGATGGCATACACAA |
| **PP2A-HA-F^b^** | ATGtacccatacgatgttcctgactatgcgTTGAGCAAGACTCTAACGCAG |
| **PP2A-3R** | GGAAGCGGACCTTAAGCG |
| **PP2A-3F-HA^b^** | CAGCCTAGGCAGGCCCGCAGCACGCCTGTGACGCAGCCATGtacccatacgatgttcctg |
| **PP2A3-Y313Δ-F** | GTG CCC GAC TTC CTT TAA GCG C |
| **PP2A3-Y313Δ-R** | GCG CTT AAA GGA AGT CGG GCA C |
| **PP2A3-L315A-F** | GAC TAC TTC GCC TAA GCG CGC |
| **PP2A3-L315A-R** | GCG CGC TTA GGC GAA GTA GTC |
| **PP2A3-L315Δ-F** | CCC GAC TAC TTC TAA GCG CGC |
| **PP2A3-L315Δ-R** | GCG CGC TTA GAA GTA GTC GGG |
| **PP2A3-V310T-F** | CGC TCC ACG CCC GAC TAC |
| **PP2A3-V310T-R** | GTA GTC GGG CGT GGA GCG |
| **PP2A3-YFLΔ-F** | GTG CCC GAC TAA GCG CGC |
| **PP2A3-YFLΔ-R** | GCG CGC TTA GTC GGG CAC |
| **PP2A3-inf-3F** | GCC TGT CGC CCT CCA TGG |
| **PP2A3-inf-3R** | GCT TTG TCT CCC ATT ATG CAC GTG |
| **PP2A3-HA-short^b^** | ATGtacccatacgatgttcctg |
| **PP2A3-4R** | CGAACATGCGTGACATTCTG |
| **PP2A3-7F** | GCAGTTCCACGACCTGATG |
| **PP2A3-3R** | GGAAGCGGACCTTAAGCG |

a. Underlined sequences are sequences from the pBlueScript vector used for cloning of PP2A3.

b. Lower case letters are sequences from the HA epitope tag.
